# Small Extracellular Vesicle and Total Lipid Profiles Provide a Diagnostic Advantage Over CA‐125 Across Ovarian Cancer Subtypes

**DOI:** 10.1002/jev2.70348

**Published:** 2026-09-01

**Authors:** Shikha Rani, Dominic Guanzon, Andrew Lai, Katherin Scholz‐Romero, Lewis Perrin, Rohan Lourie, Vaibhavi Joshi, Amy E. McCart Reed, Kaltin Ferguson, Aase Handberg, Andreas Möller, Carlos Salomon

**Affiliations:** ^1^ Translational Extracellular Vesicles in Obstetrics and Gynae‐Oncology Group Frazer Institute Faculty of Health, Medicine and Behavioural Sciences The University of Queensland Herston Queensland Australia; ^2^ UQ Centre for Extracellular Vesicle Nanomedicine Faculty of Health, Medicine and Behavioural Sciences The University of Queensland Herston Queensland Australia; ^3^ Mater Research Institute Translational Research Institute The University of Queensland Woolloongabba Queensland Australia; ^4^ Frazer institute Faculty of Medicine The University of Queensland Herston Queensland Australia; ^5^ Department of Clinical Biochemistry Aalborg University Hospital Aalborg Denmark; ^6^ Department of Otorhinolaryngology Faculty of Medicine The Chinese University of Hong Kong Sha Tin Hong Kong SAR China; ^7^ JC STEM Lab Li Ka Shing Institute of Health Sciences Faculty of Medicine The Chinese University of Hong Kong Sha Tin Hong Kong SAR China

**Keywords:** biomarkers, lipidomics, lipids, ovarian cancer, small extracellular vesicles

## Abstract

Ovarian cancer remains the most lethal gynaecological malignancy, largely due to late‐stage diagnosis and the limited sensitivity of CA‐125 for borderline and low‐grade tumours. Dysregulated lipid metabolism may provide complementary diagnostic information. This study aimed to develop lipid‐based biomarkers to improve the differential diagnosis of adnexal masses. We profiled total plasma and small extracellular vesicle (sEV) lipids in women with benign, borderline and malignant ovarian tumours, using healthy women as controls. Solid‐phase extraction demonstrated superior reproducibility, lipid‐class specificity and sEV lipid enrichment compared with liquid‐liquid extraction. Combined with CA‐125, both plasma and sEV lipid biomarkers outperformed CA‐125 alone for distinguishing borderline and low‐grade serous ovarian cancer from benign tumours (accuracy up to 0.89 vs. 0.67). In patients with normal CA‐125 levels, sEV lipids provided the greatest diagnostic advantage, distinguishing invasive and borderline tumours from benign adnexal masses (AUROC up to 0.82). Generative artificial intelligence was used to simulate biomarker performance in synthetic cohorts for differential diagnosis (*n* = 10,000) and screening (*n* = 100,000). For differential diagnosis, sEV lipids combined with CA‐125 outperformed plasma lipids for identifying endometrioid and mucinous ovarian cancer from benign adnexal masses. For screening, sEV lipids alone achieved accuracies of 0.91 and 0.94 for benign conditions and high‐grade serous ovarian cancer, respectively. Simulations restricted to patients with normal CA‐125 levels confirmed that sEV lipids provided the highest discriminative performance for low‐grade serous ovarian cancer (AUROC up to 0.74), demonstrating their potential where CA‐125 alone fails. To the best of our knowledge, this is the first study to directly compare matched plasma and sEV lipid profiles across ovarian cancer histotypes, demonstrating a diagnostic advantage for sEV lipids—particularly for borderline, low‐grade serous and mucinous tumours, supporting their integration alongside CA‐125 in future differential diagnosis and screening strategies.

## Graphical abstract

Small extracellular vesicle (sEV) lipid profiles outperform CA‐125 across ovarian cancer histotypes, achieving accuracy up to 0.73 in differential diagnosis and 0.94 in screening. In patients with normal CA‐125 levels, sEV lipids maintain discriminative performance where CA‐125 alone fails.

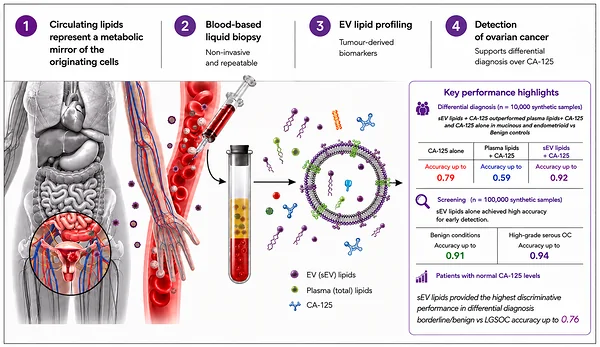

**Abbreviations**
AUROC: Area under the receiver operating characteristic curve
AUPRC: Area under the precision-recall curve
BUME: butanol methanol
CDPDAG: 3‐citidine diphosphate DAG
CE: cholesterol ester
Cer: ceramide
CerP: ceramide‐phosphate
CerPE: ceramide phosphoethanolamine
CL: cardiolipin(or diphosphatidylglycerol)
DAG: diacylglyceride
DGDG: digalactosyl DAG
DGMG: digalactosyl MAG
DGPP: DG pyrophosphate
DMPE: dimethyl PE
EOC: epithelial ovarian cancer
GD1: disialoganglioside GalGalNAc
GD2: disialoganglioside GalNAc
GD3: disialoganglioside
GlcdE: glycidol ester
GM1: monosialoganglioside GalGalNAc
GM2: monosialoganglioside GalNAc
GM3: monosialoganglioside
GT1: trisialoganglioside GalGalNAc
GT2: trisialoganglioside GalNAc
GT3: trisialoganglioside
Hex2Cer: dihexosylceramide
Hex3Cer: trihexosylceramide
HexCer: hexosylceramide
HGSOC: high grade serous ovarian cancer
IPC: Inositolphosphorylceramide
LGSOC: low grade serous ovarian cancer
LLE: liquid‐liquid extraction
M(IP)2C: mannosyl‐inositolPceramide
MADAG: monoalkyl DAG
MAG: monoacylglyceride
MGMG: monogalactosyl MAG
MIPC: mannosyl‐inositolPceramide
MMPE: monomethyl PE
MTBE: methyl‐tert‐butyl ether
NAPE: N‐acyl PE
NTA: Nanoparticle tracking analysis
OAHFA: O‐hydroxy fatty acid
PA: phosphatidic acid
PC: phosphatidylcholine
PE: phosphatidylethanolamine
PG: phosphatidylglycerol
PI: phosphatidylinositol
PIP: PI phosphate
PIP2: PI biphosphate
PIP3: PI triphosphate
PS: phosphatidylserine
SEC: Size exclusion chromatography
sEVs: small extracellular vesicles
SGalCer: sulphatides
SHAP: SHapley Additive exPlanations
SM: sphingomyelin
SPE: solid‐phase extraction
SPV: sulfo‐phospho‐vanillin
SQDG: sulphoquinovosyl DAG
TAG: triacylglyceride

## 1 Introduction

Despite decades of research into the aetiology and biology of epithelial ovarian cancer (EOC), it remains the most lethal gynaecological malignancy, largely because most patients are diagnosed at advanced stages (Lheureux et al. 2019). Approximately 75% of women with high‐grade serous ovarian carcinomas (HGSOC), the most prevalent and aggressive EOC subtype, present with non‐specific symptoms and are diagnosed late. At this stage, the 5‐year survival rate is approximately 32%, whereas if detected when the tumour is still confined to the ovaries, survival rates exceed 80% (Peres et al. 2018). In contrast, non‐high‐grade serous carcinomas (including low‐grade serous ovarian carcinoma (LGSOC), mucinous, clear cell and endometrioid carcinomas) generally grow more slowly and are associated with better prognoses (Kroeger and Drapkin 2017). Borderline tumours, which lack stromal invasion, also exhibit less aggressive behaviour compared to both high‐ and low‐grade serous ovarian cancers (Brown et al. 2007).

CA‐125 is currently the standard biomarker for ovarian cancer, particularly effective in detecting advanced‐stage disease (Sheehan et al. 1989, Niloff et al. 1984). However, even across all stages of serous ovarian cancer, CA‐125 demonstrates limited sensitivity and specificity, failing to reliably identify many malignant cases (Kolwijck et al. 2009). Its performance is especially poor in benign, borderline and non‐high‐grade serous subtypes (Buas et al. 2021, Moss et al. 2005). Furthermore, CA‐125 lacks the accuracy required for screening and has not been shown to reduce mortality or improve outcomes (Menon et al. 2009, 2021). In contrast, liquid biopsy approaches, such as lipidomic profiling of plasma and small extracellular vesicles (sEVs), offer promising advantages for early detection, disease monitoring and personalised risk stratification, potentially enabling earlier intervention and improved clinical outcomes (Feeney et al. 2020, Chang et al. 2019, Rani et al. 2023).

Extracellular vesicles (EVs) are membrane‐bound nanoparticles released by most cell types and are abundant in body fluids, where they can be captured non‐invasively as a form of liquid biopsy (Tamura et al. 2021). EVs transfer lipids, proteins and nucleic acids between cells, and are now recognised as active mediators of tumour growth, metastasis and remodelling of the tumour microenvironment, including in ovarian cancer (Rani et al. 2023, Vyhlídalová Kotrbová et al. 2024, Guanzon et al. 2026, Becker et al. 2016). Growing evidence across multiple cancer types supports the concept that EV‐associated lipids carry disease‐specific information and can serve as non‐invasive cancer biomarkers. In breast cancer, EVs derived from plasma and metastatic cell lines are enriched in distinct sphingolipids, glycerophospholipids and diacylglycerols that stratify molecular subtypes and disease stages (Dorado et al. 2024, Nishida‐Aoki et al. 2020, Liu et al. 2023), and in HR‐positive metastatic breast cancer patients, track response to CDK4/6 inhibition (Richard et al. 2024). Similar patterns have been reported in colorectal cancer, where EV lipid profiles show disease‐specific alterations across cell lines and plasma samples, with changes in sphingolipids, phospholipids, glycolipids and sterol lipids distinguishing patients from controls and reflecting disease progression and metastatic status (Lydic et al. 2015, Bestard‐Escalas et al. 2021, Elmallah et al. 2022, Eylem et al. 2020). In prostate cancer, urinary and cell‐derived EVs containing specific phosphatidylserine (PS 18:1/18:1), sphingolipids, sterol lipids differentiate malignant from non‐tumourigenic states and have been proposed as potential prostate cancer biomarkers (Llorente et al. 2013, Brzozowski et al. 2018, Skotland, Ekroos, et al. 2017). Lipidomic profiling of exosomes has also identified candidate lipid signatures in pancreatic, lung and hepatocellular carcinoma, often involving recurrent alterations in phospholipids, sphingolipids, lysophospholipids and sterol lipids (Tao et al. 2019, Fan et al. 2018, Sanchez et al. 2021). In ovarian cancer, exosomes from SKOV‐3 cells show enrichment of lysophospholipids, zymosterols, cholesterol esters and GM3 (Cheng et al. 2020), whereas plasma of women with suspected malignancy had enrichment of phosphatidylserine, providing initial proof‐of‐concept that EV lipid composition reflects ovarian tumour biology and may support differential diagnosis (Lea et al. 2017). This demonstrates that lipidomic profile from patient's biofluids and biofluid‐derived sEVs could detect the lipid signatures independent of CA‐125, adding complementary diagnostic value, particularly in cases where CA‐125 levels are low or ambiguous. This could help in cancer screening or diagnosing early‐stage disease that might not appear on conventional scans yet, which subsequently contribute to the patient's early access to treatment.

In the initial phase of this study, we systematically evaluated four liquid‐liquid extraction (LLE) methods (Bligh‐Dyer, Folch, MTBE, BUME) and two solid‐phase extraction (SPE) approaches (cartridges and 96‐well plates) to optimise lipid extraction from plasma and sEVs. We hypothesised that tumour‐driven lipid alterations captured in circulating plasma and/or sEV lipid profiles would distinguish malignant from benign and borderline ovarian lesions more accurately than CA‐125, including in subtypes where CA‐125 performs poorly. The primary aim was therefore to develop lipid‐based biomarkers for the differential diagnosis of adnexal masses, and case–control discrimination (ovarian cancer vs healthy women) was a secondary aim. We profiled plasma and matched sEVs in a clinically diverse cohort and directly compared plasma lipids, sEV lipids, CA‐125 and combined lipid + CA‐125 models.

## 2 Materials and Methods

### 2.1 Study Participants and Design

This study was performed in accordance with the Declaration of Helsinki. The overall analysis was approved by the Ethics committee of the University of Queensland and Mater Hospital (2023/HE002226), Human Research Ethics Committees at The University of Queensland (2005000785) and Royal Brisbane and Women's Hospital (RBWH 2005/022). All patients included in the clinical trials provided written informed consent. All experimental procedures were conducted within an ISO17025 accredited (National Association of Testing Authorities, Australia) research facility. All data were recorded within a 21 Code of Federal Regulation (CFR) part 11 compliant electronic laboratory notebook (Lab Archives, Carlsbad, CA 92008, USA).

Human Plasma (K2EDTA, Gender‐pooled, platelet poor; cat No. HUMANPLK2‐0104117, BIOIVT) was used for the lipid extraction LLE and SPE method optimization. Patient plasma samples and clinical data were obtained from the Mater Women's Cancer Biobank (Brisbane, Australia) and the Brisbane Breast Biobank. In this study, seven groups of participants were included: healthy controls (*n* = 38), benign ovarian lesions (*n* = 22), borderline ovarian tumours (*n* = 6), high‐grade serous ovarian carcinoma (HGSOC, *n* = 11), low‐grade serous ovarian carcinoma (LGSOC, *n* = 5), endometrioid carcinoma (*n* = 9) and mucinous carcinoma (*n* = 5). Throughout the manuscript, the term ‘invasive ovarian cancer’ (OVCA) denotes the combined group of histologically confirmed invasive epithelial ovarian carcinomas (HGSOC, LGSOC, endometrioid and mucinous; total *n* = 30), comprising both early‐stage (FIGO I) and advanced‐stage (FIGO III–IV) disease. The control group refers exclusively to healthy women; benign and borderline lesions are reported as separate diagnostic groups. Healthy controls were women undergoing gynaecological surgery for non‐ovarian, non‐malignant indications and with no history of ovarian malignancy. Benign lesions comprised serous cystadenoma/adenofibroma with or without surface papilloma, simple ovarian cysts, fibroma, leiomyoma, mucinous cystadenoma/adenofibroma, endometriosis, struma ovarii and abscess. Borderline tumours included serous borderline tumours (including a micropapillary variant) and mucinous borderline tumours. Whole blood was collected by venepuncture into ACD‐A tubes, centrifuged at 2500 × *g* for 15 min at 4°C, and the supernatant re‐centrifuged at 3000 rpm for 15 min at 4°C. Plasma was stored at –80°C until analysis.

### 2.2 Isolation of Small EVs by Size Exclusion Chromatography (SEC)

sEVs were isolated from plasma samples of ovarian cancer, benign borderline patients and control women using two preclearing steps followed by SEC. A 120 µL of plasma sample was centrifuged at 1500 x *g* for 10 min at 4°C and supernatant was collected. Further, obtained supernatant was centrifuged at 10,000 x *g* for 10 min at 4°C to remove large EVs. A 100 µL of plasma sample was used to isolate the sEVs and 10 µL crude plasma was kept for lipid extraction directly. A 100 µL of plasma sample was diluted five times with phosphate saline buffer (PBS) and SEC was performed for isolating the sEV using qEV Gen2 70 nm pore size columns and automated fraction collector setup by Izon Science Ltd. The column was flushed with two columns of PBS before loading the supernatant obtained after centrifugation of 10,000 x *g*. First three fractions of 400 µL each were collected per sample. The column was washed by running through 17 mL PBS after each 8.5 mL 0.5 M NaOH cleaning and stored in 0.05% ProClin200 storage solution.

For immunoblotting and cryo‐TEM analysis, 500 µL of pooled plasma samples from each ovarian cancer, benign‐borderline and control group was used to isolate the sEVs by collecting first three fractions. Three fractions were combined and concentrated to 100 µL using 100 kDa MWCO Amicon Ultra centrifugal filters. The relative particle and protein concentrations were measured for pooled fraction and used for immunoblotting and cryo‐TEM analysis.

### 2.3 Nanoparticle Tracking Analysis (NTA)

Particle concentration and their size distribution for sEVs obtained from plasma samples of each participant was determined by NTA using NanoSight NS500 (NTA 3.4‐Sample Assistant Build 3.4.4‐SA) (Nanosight Ltd, Malvern Instrument Ltd, Malvern, Worcestershire, UK) configured with a Blue 405 nm laser and high sensitivity scientific CMOS camera. Samples were diluted in particle free PBS to an acceptable concentration, recommended by manufacturer (optimal ∼50 particles per image) before loading to NTA system. Samples were vortexed before introducing into chamber (temperature = 25°C and viscosity = 0.89 cP) and the camera level was set to 13 with capture duration of 60 s. The captured videos (five videos/sample) were then processed and analysed to obtain mean, mode particle size with concentration average (particle/mL). Further, size distribution curve was extrapolated using GraphPad Prism Version 9.4.1 (681).

### 2.4 Protein Quantification

The protein concentration was analysed using the pierce BCA Assay kit (Thermo Fisher) following manufacturer's instructions. The sEV samples were diluted in 1:1 ratio and then compared in triplicates against serially diluted bovine serum albumin (BSA) as standard. The SPECTROstar Nano microplate reader by BMG Labtech, Ortenberg, Germany was used to measure the absorbance at wavelength of 562 nm for standard and fractions containing particles obtained by size exclusion chromatography. BCA standard curve was plotted and protein concentration (µg/mL) for each fraction were extrapolated from the curve.

### 2.5 Immunoblotting Analyses

Equal amount of proteins were loaded for sEVs and plasma samples. Each protein sample was mixed with 10× Bolt sample reducing agent and 4× Bolt LDS sample buffer and heated at 70°C for 10 min. Proteins were resolved using Bolt 4%–12% Bis‐Tris‐Plus gel on SDS‐polyacrylamide gel electrophoresis cassette at 180 V for 40 min and transferred to Wet Immobilon‐FL PVDF membrane (pore size 0.45 µm) at 100 V for 1 hour. The membrane was blocked for 1 hour in Intercept blocking buffer (LI‐COR Biosciences) followed by probing with primary antibodies. Proteins were detected by incubation of membranes with following primary antibodies: Rabbit mAb to CD9 (cat. No. 13174S; 1:1000 dilution; Cell Signalling Technology Inc.), rabbit mAb to CD81(cat. No. 56039S; 1:1000 dilution; Cell Signalling Technology Inc.), rabbit monoclonal anti‐ALIX (cat. No. ab186728; 1:1000 dilution; Abcam), rabbit mAb to flotillin‐1(cat. No. 18634S; 1:1000 dilution; Cell Signalling Technology Inc.), rabbit recombinant monoclonal antibody to ApoA1(cat. No. ab52945; 1:1000 dilution; Abcam) and bovine serum Albumin antibody (cat. No. ab192603;1:1000 dilution; Abcam) in Intercept antibody dilution buffer (LI‐COR Biosciences) overnight at 4°C. Following two 5 min washes with 1× washing buffer containing TBS with 0.1% Tween‐20 (TBS‐T), membranes were incubated in IRDye800‐conjugated goat anti‐rabbit IgG secondary antibodies (LI‐COR Biosciences) (1:10,000 dilution). The blots were then washed three times with 1× washing buffer TBS‐T for 10 min each. Proteins were then visualized by scanning the blots on ChemiDoc with both 700‐ and 800‐nm channels.

### 2.6 Cryogenic Transmission Electron Microscopy (Cryo‐TEM)

sEV‐samples were imaged by cryo‐EM at centre for microscopy and microanalysis, The University of Queensland (UQ). The purified sEV‐samples were prepared using Leica EM GP2 robotic vitrification system, under controlled temperature of 22°C and relative humidity of 95%. Three microlitre of sEVs was applied onto a carbon coated perforated formvar film supported on a 200 mesh copper TEM grid. Any excess solution was automatically blotted for a period of 3–3.5 s and rapidly plunged into liquid ethane near to its freezing point (–182.8°C). The prepared grids were stored under liquid nitrogen prior to imaging. The imaging of samples was done on Jeol Cryo ARM200 (JEM‐Z200FSC) transmission electron microscopy equipped with a cold field emission gun and in‐column omega energy filter in a frozen hydrated state at –176°C. The capturing of images was done at zero energy loss at the acceleration voltage of 200 kV and filter setting of 20 eV. Image recording was done under low‐dose conditions for minimal exposure using SerialEM software and Gatan K2 direct detector camera.

### 2.7 Enzyme‐Linked Immunosorbent Assay (ELISA)

CA‐125 levels in all plasma and sEV samples were quantified using the Human CA‐125/MUC16 Quantikine enzyme‐linked immunosorbent assay (ELISA) kit (catalogue number DCA 125; R&D Systems, Minneapolis, MN), according to manufacturer's instructions. Optical density (OD) was measured at 450 nm with a reference at 540 nm using SPECTROstar Nano microplate reader by BMG Labtech, Ortenberg. For quality control, Lyphochek Tumour Marker Plus low, medium and high CA‐125 control sera (catalogue number 548X; Bio‐Rad, Hercules, CA) were diluted 1:10 in 1× Dulbecco's phosphate‐buffered saline (DPBS, Gibco catalogue number 2858899; Thermo Fisher Scientific). Samples exceeding the assay's detection range (5–320 U/mL), as determined from the standard curve, were diluted 1:100 in DPBS and reanalysed to obtain accurate measurement.

### 2.8 Sulfo‐Phospho‐Vanillin (SPV) Lipid Quantification Assay

Lipid standard from Lipid Assay kit (unsaturated fatty acids Cat no. ab242305; Abcam) was used to generate a standard curve for lipid quantification. For each sample, 15 µL of SEC fractions or standard solutions were transferred into 1.5 mL microcentrifuge tubes and incubated uncovered at 90°C for 30 min to completely evaporate the organic solvent. Tubes were subsequently cooled at 4°C for 5 min. Afterward, 150 µL of 18 M sulphuric acid (Cat no.1.00714; Merck) was added to each tube, followed by incubation at 90°C for 10 min. After incubation, samples were again cooled at 4°C for 5 min. Subsequently, 100 µL of each reaction mixture was transferred to the clear 96‐well microplate and the background absorbance was measured at 540 nm using SPECTROstar Nano microplate reader by BMG Labtech, Ortenberg, Germany. Then, 100 µL of 1 mg/mL vanillin reagent (Cat no. V1104; Merck) prepared in 17% phosphoric acid (Cat no.345245; Merck) was added to each well, and the plate was incubated at 37°C for 60 min. Following to colour development, absorbance was recorded at 540 nm. Unsaturated fatty acid concentrations in SEC fractions were determined by extrapolation from standard curve and expressed in mg/dL.

### 2.9 Liquid‐Liquid Extraction (LLE) Methods

Four LLE methods (Bligh‐Dyer, Folch, MTBE and BUME) were evaluated in parallel using 100 µL of plasma or sEV input. In brief, samples were mixed with the appropriate solvent system (chloroform/methanol 1:1 for Bligh‐Dyer; chloroform/methanol 2:1 for Folch; MTBE/methanol 2:1 followed by re‐extraction with MTBE/methanol/water (10:3:2.5 v/v) for MTBE; butanol/methanol 1:1 for BUME), vortexed and incubated on ice, phase‐separated, and the organic phase collected, dried under nitrogen, reconstituted in 100 µL ethanol, and centrifuged before LC‐MS analysis. Full step‐by‐step protocols for each LLE method, including incubation times and centrifugation parameters, are provided in Supporting Information Methods.

### 2.10 Lipid Extraction—Solid Phase Extraction (SPE)

A 100 µL BioIVT plasma samples were used to directly extract the lipids using bond elute SPE cartridge and plate for method optimization. The sEVs isolated from plasma were processed similarly as the plasma samples for lipid extraction. The plasma and sEV samples from patients were spiked in with 5 µL UltimateSPLASH ONE Mass Spec Standard (cat No. 330820, Avanti POLAR LIPIDS) (2× diluted) to allow for quality control. All reagents were MS grade or equivalent. Lipid extractions were performed using Bond Elut Lipid Extraction SPE 2 mL 96‐well plates (cat No. 5610‐2043, Agilent) and SPE cartridge (cat No. 5610‐2041, Agilent) for plasma and sEV samples for method optimization. The SPE 2 mL 96‐well plates were used for lipid extraction from clinical samples. The workflow for lipid extraction using SPE cartridge or plate includes four steps‐ protein precipitation, sample loading, sample washing and lipid elution. A plasma or sEV samples were mixed with ice‐cold 900 µL of ACN/MeoH (99:1, v/v) for protein precipitation and vortexed for 30 s followed by sonication for 10 min on ice. The sonication was done to release the protein precipitation from sample to get improved efficiency of lipid extraction. The obtained homogenate was transferred to Bond Elut Lipid extraction SPE 96 well plate or cartridge and steadily eluted under gravity for sufficient interaction time between lipid compounds and sorbent for the efficient retention on sorbent, further flowthrough was discarded. The SPE plate or cartridge was then washed twice with 1 mL of ACN/water (9:1, v/v) under low pressure and flowthrough was discarded. Further, plate was dried under high pressure (6–9 psi) and waste reservoir was removed. The 2 mL glass coated collection plate or glass tube was placed under SPE plate or cartridge and lipids were eluted twice by 1 mL of chloroform/MeOH (1:1, v/v) with gravity of low pressure and high pressure (6–9 psi) was applied at the end to dry sorbent. The obtained eluent was then dried with N2 at room temperature. The dried lipid residue was reconstituted into 100 µL of 100% ethanol with 2 min vortex and 10 min of sonication at room temperature for complete redissolving of dried lipid residues. The 50 µL of lipid samples were then sent to LC‐MS facility, UQ Frazer Institute, Brisbane, Australia for lipidomic analysis.

### 2.11 Lipid Classes LC‐MS Analysis and Quantitative Data Processing

A 12 µL of sample were directly infused using 1:1 methanol: water with 0.1% formic acid as mobile phase at 2.5 µL/min flow rate using Eksigent LC200 micro liquid chromatography (LC) system. Tau acid was injected for internal calibration to maintain mass accuracy over time whereas the X500B was used for calibration in positive mode ensuring the accurate mass detection across a wide range of lipids prior to the analysing samples. SCIEX 5600– qTOF mass spectrometer was used to acquire mass spectra in positive and negative modes. TOFMS were acquired from 100 to 2000 Da (+) and 300 to 2000 Da (–), parameters of mass spectrometry were as follows: ion spray voltage, 5500 V (+) and 4500 V (−); curtain gas of 30 PSI; declustering potential, 100 V (+) and −100 V (−); collision energy, 15 V (+) and −10 V (−); and interface heater temperature, 250°C (+) and 250°C (−). IDA analysis (MSMS spectra) were acquired in high sensitivity mode from 50 to 2000 Da. The collision energy spread was equal to 30 in both positive and negative ion modes. The output of the SCIEX 5600– qTOF mass spectrometer analysis was imported to Lipidview, SCIEX software for molecular identification and quantification using m/z values and peak area. The numerical matrix file was generated for lipid ID and the observed peak area of the lipid for each sample. Although the UltimateSPLASH ONE internal standard mix was spiked during sample preparation, these standards were not utilized in downstream data processing due to limitations in spectral annotation during untargeted analysis. Untargeted lipidomic profiling was performed and lipid species were annotated post‐acquisition using LipidView software (Sciex); lipid classes were not preselected prior to analysis but reflect those detectable under the applied LC‐MS conditions and within the software's reference database. The full list of lipid classes selected for analysis within the software is provided in Table S1.

### 2.12 Generative AI‐Based Data Augmentation

We used a generative artificial intelligence (GAI)‐based data augmentation workflow to improve model performance, as previously described (Joglekar et al. 2025). Briefly, participant lipidomic data were used to fit a Gaussian Copula Synthesizer implemented in the Synthetic Data Vault (SDV) Python library to generate two synthetic cohorts reflecting distinct clinical scenarios. For differential diagnosis, a cohort of 10,000 women with an adnexal mass was simulated, comprising high‐grade serous carcinoma (HGSOC; *n* = 2100), endometrioid carcinoma (*n* = 300), mucinous carcinoma (*n* = 150), low‐grade serous carcinoma (LGSOC; *n* = 150), borderline tumours (*n* = 1300) and benign tumours (*n* = 6000). For screening, a cohort of 100,000 symptomatic women was simulated using a mid‐range ovarian cancer prevalence of 0.5%, comprising ovarian cancer cases (*n* = 500), benign adnexal masses (*n* = 3000; prevalence 3%) and controls without an adnexal mass (*n* = 96,500). Synthetic data quality was evaluated using the evaluate quality function from the SDV framework, which compares the statistical properties of synthetic data against the original dataset across three metrics: the Overall Quality Score (OQS, overall similarity between real and synthetic cohorts, scaled 0%–100%), Column Shapes Score (CSS, similarity of marginal distributions of individual variables) and Column Pair Trends (CPT, preservation of pairwise relationships and correlations between variables). For the sEV cohort, the synthetic data achieved an OQS of 59.9%, a CSS of 22.4% and a CPT score of 97.3%, indicating strong preservation of inter‐variable relationships despite limited reproduction of individual variable distributions. For the plasma cohort, the OQS and CPT score were both 50.0%; the CSS could not be calculated by SDV (Table S2). These results indicate that the synthetic cohorts retained varying degrees of the statistical characteristics of the original data, with stronger preservation of multivariate structure observed in the sEV cohort. Importantly, synthetic cohorts were used only for downstream performance evaluation and generalisability assessment of models trained on real‐world data; synthetic samples were not used to train the classifiers. Because synthetic samples are derived from the original limited dataset, they cannot capture the full biological and clinical variability of independent cohorts and cannot substitute for external validation in real‐world prospective populations.

### 2.13 Statistical Analysis

A lipidomics dataset was processed by applying an inclusion criterion, sorting only those lipid species with peak areas detected in more than 30% of the samples. The filtered dataset was then log_2_(x + 1) transformed to normalize the distribution. Differential analysis between two groups was performed in R and results were visualized using a volcano plot, displays the log_2_ fold change (LogFC) and adjusted *p* values for each lipid species. The prediction model was built to distinguish control and ovarian cancer patient in an automated and systemized way. Both presence and absence of cancer as well as the counts of detected lipid species were included as input and 14 machine learning based classification algorithms such as logistic regression, random forest analysis, support vector machine‐radial kernel, Gaussian process classifier, multilayer perceptron light gradient boosting machine, K neighbours classifier, AdaBoost classifier, linear discriminant analysis, quadratic discriminant analysis, naïve Bayes, decision tree, gradient boosting, extreme gradient boosting classifier; was applied from scikit‐learn package in Python. Further, 70% data was trained and applied the model with high area under precision‐recall curve (AUPRC) to the remaining 30% for testing set and the best‐performing model was selected. Area under the receiver operating characteristics (AUROC) curve was determined to measure the accuracy of classification model generated with features (lipid species) and Brier score in python Pyroc 0.1.1 package. For each biomarker panel and comparison, the 14 machine‐learning classifiers were trained and selected, for primary reporting, the single algorithm that achieved the highest AUROC (with favourable sensitivity, specificity and calibration) in the independent test set; performance metrics for all algorithms are provided in Table S3A. Age differences between diagnostic groups were assessed across all pairwise comparisons, and while statistically significant differences were observed in comparisons involving HGSOC and LGSOC, reflecting the known later onset of these subtypes, an age‐stratified sensitivity analysis confirmed that lipid signatures maintained strong discriminative performance across all comparisons (AUROC 0.74–1.00), with age contributing at most moderately (10%–20%) to biomarker performance (Table S3B).

## 3 Results

### 3.1 Optimisation of Lipid Extraction From Plasma and Small EVs Using SPE and LLE Methods

**FIGURE 1:**
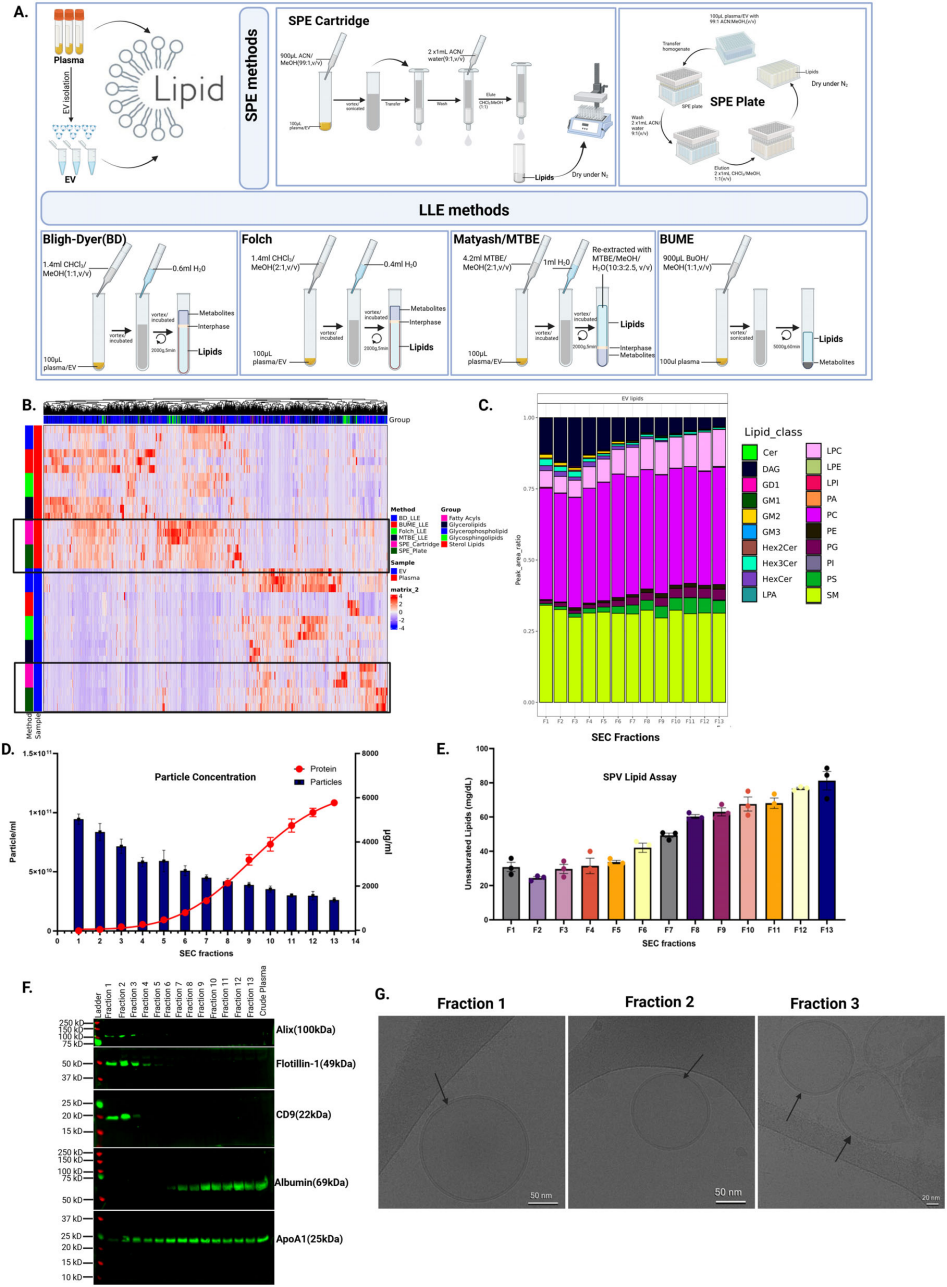
Lipid extraction method development utilizing plasma and sEV samples. (A) LLE and SPE lipid extraction method workflow for plasma and derived sEVs. (B) Hierarchical lipid clustering by LLE and SPE lipid sample preparation methods for positive and negative combined LC‐MS data in plasma and sEV samples from control individuals. Each extraction method is represented by three preparation replicates. (C) Lipid classes abundance in 13 SEC fractions distinguishing sEV (1‐3) and Non‐EV (4‐13). (D) Protein and particle concentration measurement. (E) Unsaturated lipid abundance in SEC fractions (F1‐F13) using sulfo‐phospho‐vanillin (SPV) lipid assay. (F) Immunoblotting for EV transmembrane (CD9) and cytosolic proteins (Alix and Flotillin‐1). Non‐EV markers (albumin and ApoA1) used as purity control. (G) Representative cryo‐ transmission electron microscopy (cryo‐TEM) images acquired for sEVs in SEC Fractions (F1‐F3).

We systematically compared four LLE methods (i.e., Bligh‐Dyer, Folch, MTBE and BUME) and two SPE approaches using cartridges and 96‐well plates, aiming to identify the most effective and reproducible lipid extraction method for plasma and sEV samples (Figure 1A). This optimization was designed with the plan to support future biomarker discovery studies in larger clinical cohorts, with a focus on low sample volumes and potential for automation. Total ion chromatographs (TICs) from both positive and negative ionization modes are shown in Figure S1, providing an overview of lipid profiles detected by each extraction method. To evaluate the performance of each method, lipid profiles were further analysed using hierarchical clustering based on lipid classes detected in both positive and negative LC‐MS modes. SPE methods consistently yielded more distinct and reproducible lipid profiles across replicates in both plasma and sEVs than LLE methods (Figure 1B). Principal component analysis (PCA) further confirmed the tighter clustering of SPE‐derived samples, highlighting the method's superior reproducibility and robustness (Figure S2A,B).

SPE also showed reduced variability and improved recovery of key lipid classes, including fatty acyls, glycerolipids, glycerophospholipids, glycosphingolipids and sterol lipids, compared to LLE (Figure 1B). To assess sEV‐specific lipid enrichment, plasma samples were fractionated by SEC, and lipid class distribution was analysed across fractions 1–13. Notably, SPE revealed enrichment of sEV‐associated lipid classes (e.g., PC, SM, HexCer, Hex3Cer, GM2 gangliosides and DAG) specifically in fractions 1–3, indicating successful separation of sEVs from non‐EV particles (Figure 1C).

Further characterisation of SEC fractions showed that particles and protein concentrations peaked in fractions 1–3 (Figure 1D), with a parallel trend observed for unsaturated fatty acid content (Figure 1E). Immunoblotting of classical sEV markers (CD9, Alix and Flotillin‐1) confirmed sEV enrichment in these early fractions, while non‐EV markers (Albumin and ApoA1) were either absent or minimally detected (Figure 1F). Cryo‐EM images of fractions 1–3 further validated sEV enrichment, showing vesicles with characteristic double‐membrane morphology (Figure 1G).

### 3.2 Lipid Panels Are Superior to CA‐125 in Ovarian Cancer Diagnosis

Ovarian cancer is histologically heterogeneous. Histological subtype and FIGO stage were determined from final pathology and multidisciplinary team records. The malignant cohort included HGSOC (*n* = 11; predominantly FIGO III–IV), LGSOC (*n* = 5; FIGO IA to IIIC–IV), mucinous (*n* = 5; FIGO IA–IIIC) and endometrioid (*n* = 9; mostly FIGO I, with several III–IV) carcinomas, so both early (FIGO I) and late (FIGO ≥III) disease were represented for each histotype (Table S4). Clear cell carcinoma was not included because no eligible cases were available during the recruitment period.

**FIGURE 2:**
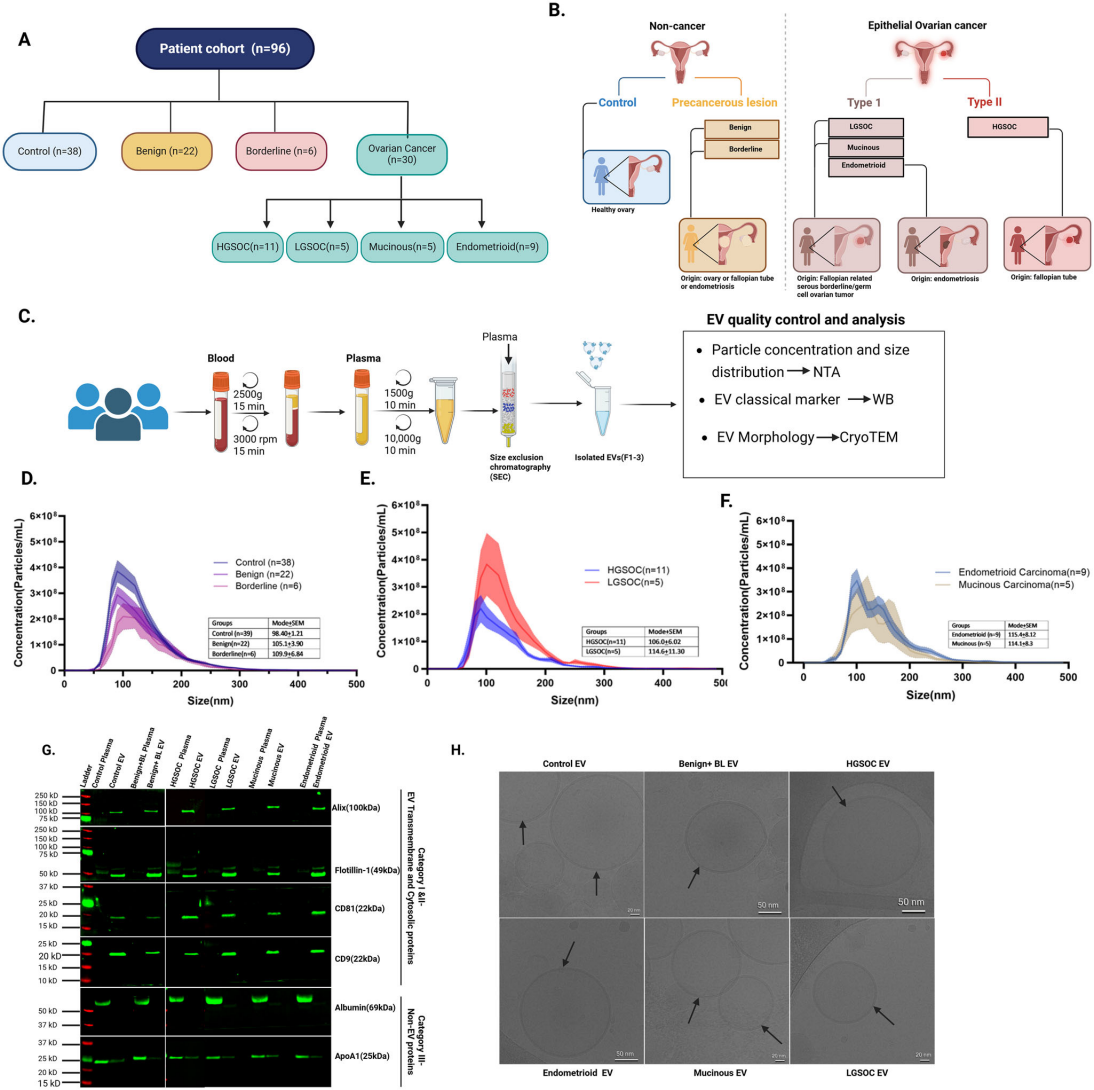
Characterization of sEVs isolated from ovarian cancer patient's plasma by size exclusion chromatography. (A) Details of patient cohort (B) Graphical representation of types of epithelial ovarian cancer (EOC). (C) Plasma‐derived sEV isolation and characterization workflow. (D), (E) and (F) Nanoparticle tracking analysis (NTA) shows the size distribution of extracellular vesicles isolated from plasma samples of control, benign, borderline, HGSOC, LGSOC, endometrioid and mucinous carcinoma patients. Particle size presented in Mode± SEM. (G) Immunoblotting for EV transmembrane (CD9 and CD81) and cytosolic proteins (Alix and Flotillin‐1). Non‐EV markers (albumin and ApoA1) used as purity control. (H) Representative cryo‐transmission electron microscopy (cryo‐TEM) images acquired for sEVs isolated from control, benign+ borderline, ovarian cancer patient's pooled plasma.

Clinical characteristics, including age at diagnosis, BMI, CA‐125 levels and lymph node status, are summarised in Table 1. Plasma CA‐125 levels differed significantly between control, benign, borderline and invasive ovarian cancer groups (*p* < 0.05), whereas other clinical variables showed no significant variation. sEVs were isolated from plasma using SEC and fractions 1–3 were pooled for downstream analysis. These sEV‐enriched fractions were characterised by size, morphology and expression of canonical EV markers, as shown in the workflow (Figure 2C). Cryo‐electron microscopy (Figure 2H) revealed bilayered, oval‐shaped vesicles, while nanoparticle tracking analysis (NTA; Figure 2D–F) showed mode particle size ranging from 98.4 ± 1.2 nm to 115.4 ± 8.1 nm, respectively. Particle mode size and concentration across groups are presented in Figure S3A,B. EV concentration varied most across groups, with endometrioid and healthy samples showing higher mean concentrations and HGSOC displaying lower, but more heterogeneous distributions. In contrast, EV size (mean and mode) was relatively stable (∼120–150 nm), consistent with small EV populations, and healthy samples showed narrower confidence intervals, indicating more homogeneous vesicle populations (Table S5). Kruskal–Wallis testing demonstrated a significant difference in EV concentration across groups (*p* = 0.046), but not in EV mean or mode size. Post hoc analysis identified higher EV concentrations in endometrioid and healthy samples compared with HGSOC, while pairwise differences were not observed for EV size metrics (Table S5). Immunoblotting confirmed enrichment of CD9, CD81, Flotillin‐1 and Alix in pooled sEV fractions (Figure 2G), whereas albumin and ApoA1 were depleted from sEVs and abundant in unfractionated plasma, supporting high preparation purity.

**TABLE 1:** Clinical characteristics of ovarian cancer patient's sample evaluated.

	Groups				
Characteristics	Control (*n* = 38)	Benign (*n* = 22)	Borderline (*n* = 6)	Invasive OVCA (*n* = 30)	*p* value
Age at the time of diagnosis (years)	52.97 ± 13.21 (32.00–77.00)	52.27 ± 15.78 (20.00–74.00)	49.67 ± 15.40 (31.00–70.00)	59.20 ± 17.58 (24.00–83.00)	0.2465 ns
Weight (kg)	N/A	74.18 ± 28.76 (47.00–161.0)	65.23 ± 1.66 (63.70–67.00)	80.34 ± 39.28 (40.00–174.0)	0.7381 ns
Height (cm)	N/A	163.4 ± 7.25 (150.0–172.0)	161.3 ± 1.52 (160.0–163.0)	160 ± 7.183 (149.0–172.0)	0.4394 ns
BMI	27.86 ± 5.05 (18.00–36.80)	27.31± 9.33 (19.00–57.80)	24.80 ± 0.78 (24.00–25.85)	32.19 ± 12.16 (15.94–60.00)	0.3176 ns
CA‐125 levels (U/mL)	10.69 ± 6.98 (3.87–37.77)	31.64 ± 56.64 (4.71–249.6)	17.47 ± 11.86 (7.04–38.32)	317.9 ± 494.5 (19.28–2112)	<0.0001^****^
Number lymph nodes dissected	N/A	N/A	2.00 ± 0.00 (2.00–2.00)	5.00 ± 6.31 (1.00–22.00)	0.5278 ns
Number lymph nodes positive	N/A	N/A	0.00 ± 0.00 (0.00–0.00)	0.41 ± 0.66 (0.00–2.00)	0.4107 ns

*Note*: Data presented as mean ± standard deviation [IQR]. Ordinary one‐way ANOVA test was used to determine difference between groups. Lymph node information was compared between the borderline and invasive OVCA groups using an unpaired *t*‐test to assess differences in the number of dissected and positive lymph nodes.

* **** *p* <0.0001, ns.

**FIGURE 3:**
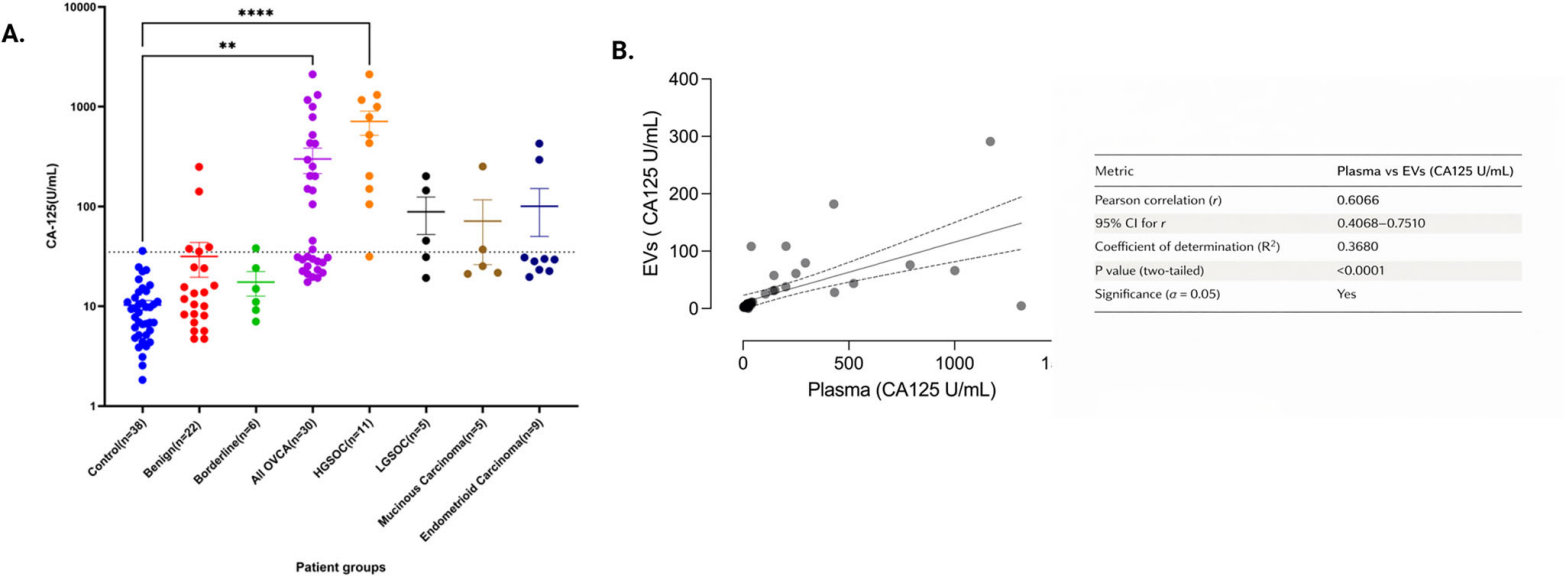
Quantification of plasma CA‐125(U/mL) and their correlation with sEV samples. (A) CA‐125 levels (U/mL) in patient groups‐control, benign, borderline, Ovarian cancer subtypes‐HGSOC, LGSOC, mucinous and endometrioid carcinoma. Data were compared using an ordinary one‐way Anova test.****p < 0.0001 ***p < 0.001, **p < 0.01, *p < 0.05. (B) The correlation between CA‐125 levels in plasma and matched sEV samples from ovarian cancer patients was assessed using Pearson's correlation coefficient. A positive correlation was observed between sEV CA‐125 and plasma CA‐125 levels (*r* = 0.60, U/mL).

CA‐125 is a well‐established biomarker for advanced‐stage ovarian cancer, particularly HGSOC, where levels are typically elevated. To assess its performance across disease stages and subtypes, we quantified plasma CA‐125 (U/mL) in healthy controls, benign cases, borderline tumours and ovarian cancer subtypes (HGSOC, LGSOC, mucinous and endometrioid). As expected, CA‐125 levels were significantly higher in HGSOC than in controls (Figure 3A). However, no significant differences were observed between controls and benign, borderline, LGSOC, mucinous or endometrioid groups (Figure 3A), highlighting the limited value of CA‐125 for early‐stage or less aggressive disease and for differential diagnosis. To address this diagnostic gap, we investigated plasma and sEV lipidomic profiles as alternative biomarkers. Because ovarian tumourigenesis is frequently associated with dysregulated lipid metabolism, these profiles may provide greater sensitivity than conventional protein markers. We also observed a strong correlation between CA‐125 levels in plasma and matched sEV samples (Pearson *r* = 0.60; Figure 3B), supporting the utility of parallel plasma and sEV analyses in biomarker development.

**FIGURE 4:**
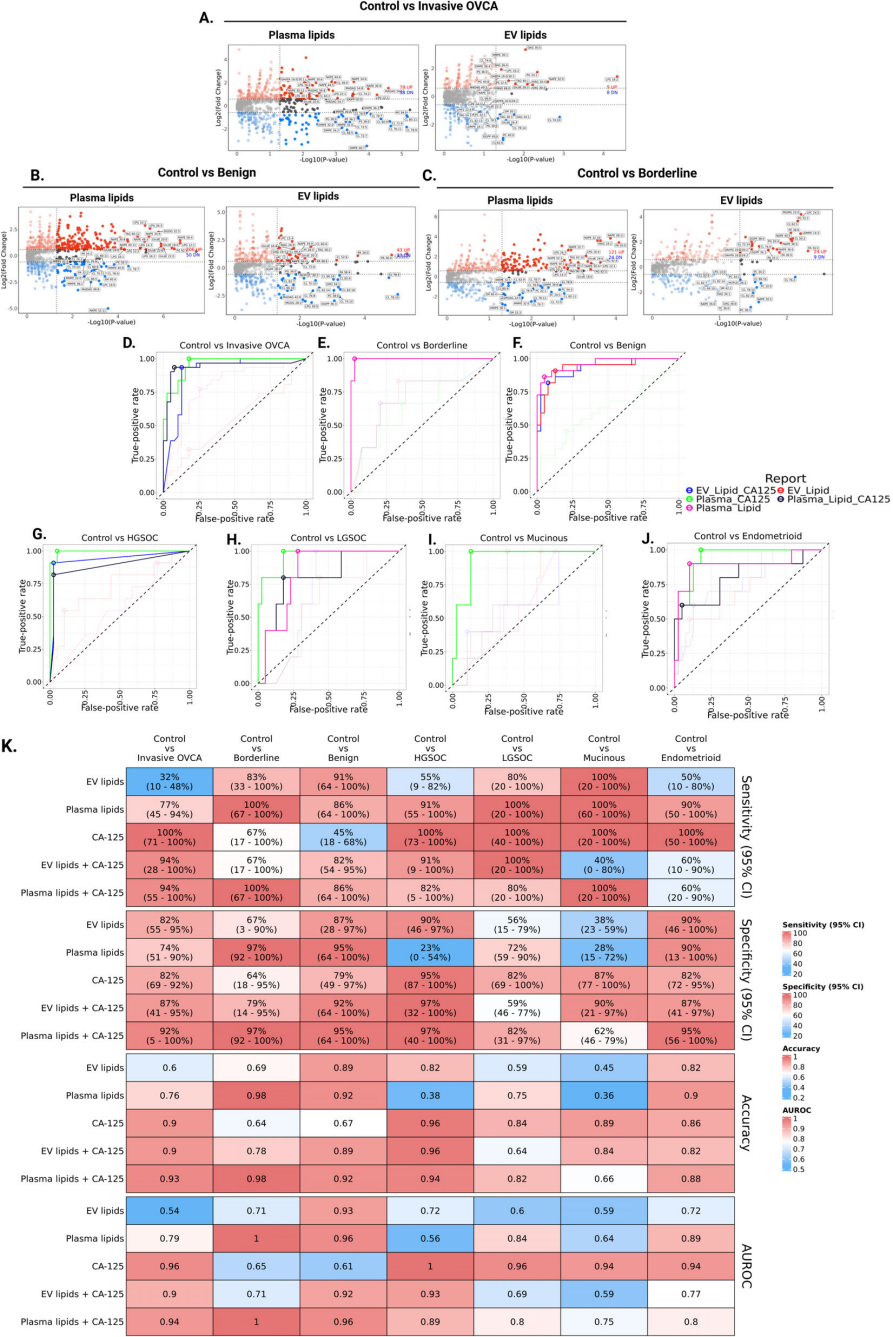
Integrated analysis of biomarker combinations performance comparing Ovarian cancer (OVCA) groups with control. Volcano plot showing differential expression of Plasma and sEV lipids in (A) Control versus Invasive OVCA, (B) Control versus Benign and (C) Control versus Borderline. Overlaid Receiver Operating characteristics (ROC) curve for the classification performance of biomarker combinations EV lipids, plasma lipids, CA‐125, EV lipids + CA‐125 and plasma+CA‐125 for (D) Control versus Invasive OVCA, (E) Control versus Borderline, (F) Control versus Benign, (G) Control versus HGSOC, (H) Control versus LGSOC, (I) Control versus Mucinous and (J) Control versus Endometrioid carcinoma. (K) Table summarizing sensitivity and specificity (95% CI), accuracy and AUROC for each combination. All model performance metrics shown in this figure are derived from the independent test cohort (30% of samples), which was not used for model training or tuning (training cohort = 70% of samples).

To characterise lipidomic alterations, we performed differential expression analysis using volcano plots to identify lipid subclasses in plasma and sEVs across cancer subtypes versus controls (Figure 4A–C, Figure S4). Plasma yielded more differentially expressed lipids than sEVs, consistent with its more heterogeneous lipid content from lipoproteins and other circulating components. Unsupervised visualisation using hierarchical clustering and UMAP revealed clear structure across sample types and clinical groups (Figure S5A,B). Plasma and sEV samples formed distinct blocks in the heatmap of all quantified lipids, and within each compartment invasive ovarian cancer, benign, borderline and control samples segregated into discrete clusters, consistent with extensive disease‐associated lipid reprogramming. In the UMAP projection, plasma and sEV samples occupied non‐overlapping regions, with invasive ovarian cancer separated from controls and benign and borderline samples typically intermediate, supporting the biological relevance of the lipid signatures.

We next evaluated diagnostic performance using receiver operating characteristic (ROC) curves and classification metrics. To minimise batch effects, plasma and matched sEV samples from each patient were block‐randomised before lipid extraction and untargeted LC‐MS. The dataset was randomly split into a discovery/training cohort (70%) and an independent test cohort (30%), enabling robust performance assessment and reducing overfitting. We assessed lipid panels (alone and combined with CA‐125) for differentiating ovarian cancer subtypes from controls. CA‐125 performed well for advanced cancers, with AUROC values of 1.00, 0.96, 0.94 and 0.94 for HGSOC, LGSOC, mucinous and endometrioid carcinomas, respectively (Figure 4G–J), and corresponding sensitivity, specificity and accuracy summarised in Figure 4K. By contrast, CA‐125 showed limited performance for borderline tumours and benign conditions (AUROC 0.65 and 0.61, respectively; Figure 4E,F,K).

Lipid panels, particularly plasma‐ and sEV‐derived signatures, performed better in these diagnostically challenging settings. For control versus borderline tumours, plasma lipids achieved an AUROC of 1.00 (sensitivity 100%, specificity 97%), and sEV lipids achieved 0.71 (sensitivity 83%, specificity 67%) (Figure 4K). For control versus benign, plasma lipids reached an AUROC of 0.96 (sensitivity 86%, specificity 95%), and sEV lipids 0.93 (sensitivity 91%, specificity 87%) (Figure 4K). These results indicate that plasma and sEV lipid profiles offer strong discriminatory power where CA‐125 is weak. Supporting performance metrics, including AUPRC and calibration curves, are shown in Figure S6A and were used when training predictive models.

Top contributing lipids distinguishing controls from borderline tumours were CL 80:2 and PA 34:0 in sEVs and CL 50:1 and NAPE 32:10 in plasma (Figures S7 and S8, Table S7). For control versus benign, key features were LPS 30:0 and CL 78:01 in sEVs and LPG 20:2 and LPC 16:0 in plasma (Figures S7 and S8, Table S7), suggesting a role for lysophospholipids in the development of non‐invasive disease.

When analysis was restricted to early‐stage disease, multimodal models performed best: sEV lipids + CA‐125 and plasma lipids + CA‐125 maintained high sensitivity for early‐stage tumours while correctly classifying nearly all controls, whereas CA‐125 alone and plasma lipids alone produced more early‐stage false‐negatives (Figure S9). Power calculations for the five biomarker panels are summarised in Table S6. Overall, sEV lipids + CA‐125 showed the highest and most consistent statistical power (mean ± SD 52.3 ± 36.2%, 95% CI 37.8%–66.8%), with several comparisons, particularly control/benign versus overall ovarian cancer and HGSOC, reaching 100% power. CA‐125 alone also showed high mean power (76.6 ± 30.5%, 95% CI 64.5%–88.7%), especially for advanced disease, but with reduced performance in borderline and mucinous subtypes. EV lipids alone and plasma lipids alone showed more variable power (means 31.0% and 42.5%, respectively), while plasma lipids + CA‐125 improved performance but remained heterogeneous across histotypes. Lower statistical power for borderline, mucinous and LGSOC comparisons is primarily driven by the small sample sizes of these subgroups (LGSOC *n* = 5, mucinous *n* = 5, borderline *n* = 6), with the biological heterogeneity likely contributing additionally. Results for these small subgroups should therefore be interpreted with caution and require confirmation in larger cohorts.

### 3.3 Lipid‐Based Biomarkers Improve Differential Diagnosis in Ovarian Histotypes

**FIGURE 5:**
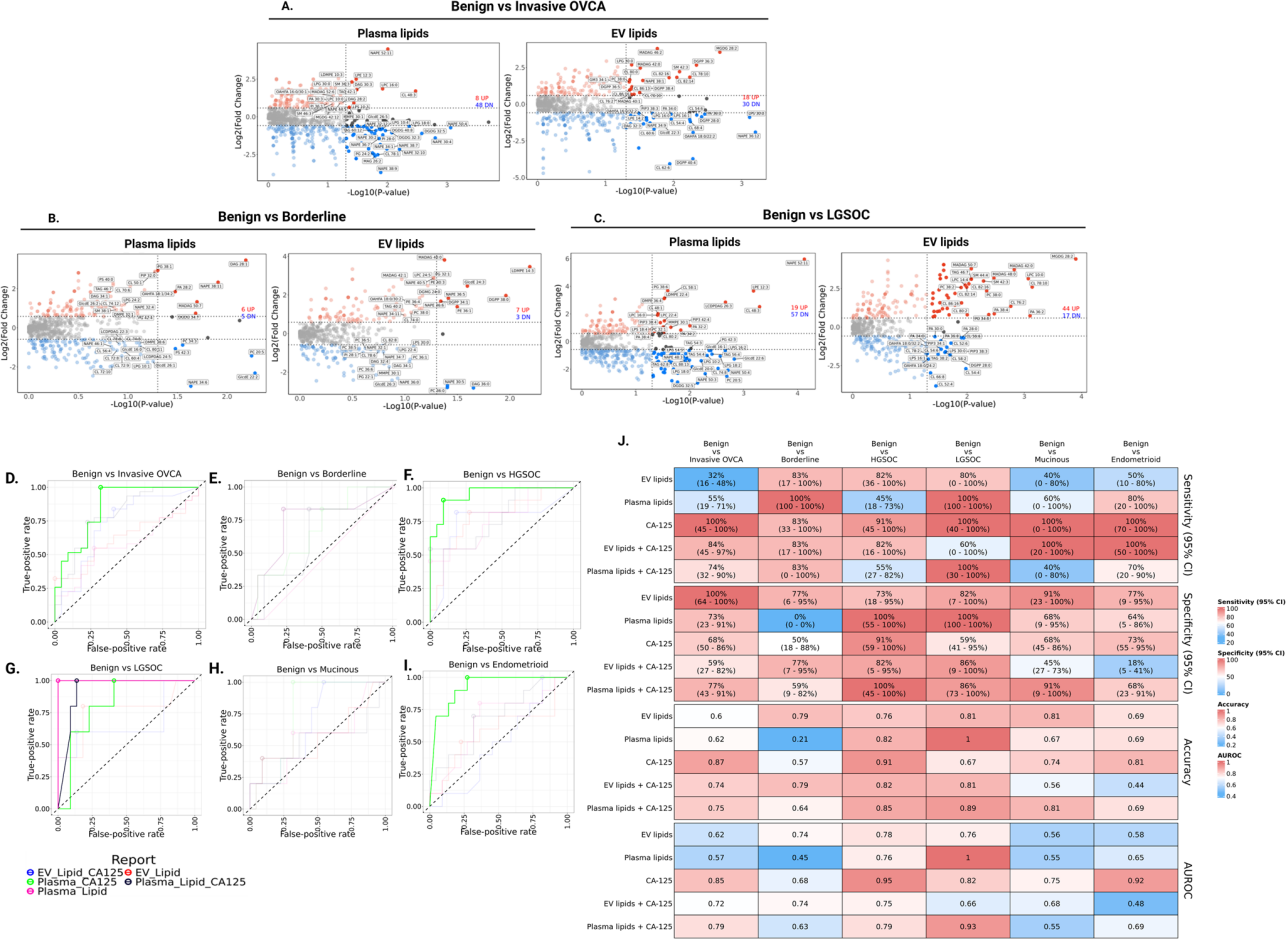
Comparative analysis of biomarker combinations comparing Benign patients with Ovarian cancer. Volcano plot showing differential expression of Plasma and sEV lipids in (A) Benign versus Invasive OVCA, (B) Benign versus Borderline (C) Benign versus LGSOC. Overlaid Receiver Operating characteristics (ROC) curve for the classification performance of biomarker combinations EV lipids, plasma lipids, CA‐125, EV lipids + CA‐125 and plasma + CA‐125 for (D) Benign versus Invasive OVCA, (E) Benign versus Borderline, (F) Benign versus HGSOC, (G) Benign versus LGSOC, (H) Benign versus Mucinous and (I) Benign versus Endometrioid carcinoma. (J) Table summarizing sensitivity and specificity (95% CI), accuracy and AUROC for each combination. All model performance metrics shown in this figure are derived from the independent test cohort (30% of samples), which was not used for model training or tuning (training cohort = 70% of samples).

Building on the control versus ovarian cancer analyses, we next evaluated sEV lipids, plasma lipids and CA‐125 for differentiating benign conditions from specific tumour subtypes, including benign versus overall invasive OVCA, benign versus borderline and benign versus individual histotypes (HGSOC, LGSOC, mucinous, endometrioid) (Figure 5). Differentially regulated lipids were visualised by volcano plots (Figure 5A–C, Figure S10A–F). Although CA‐125 remains a key marker for advanced disease (Figure 5D–I), it showed limited value for subtype‐level and differential diagnosis. sEV lipids outperformed both plasma lipids and CA‐125 in distinguishing benign from borderline tumours (AUROC 0.74; sensitivity 83%; specificity 77%; Figure 5E,J). For benign versus LGSOC, plasma lipids achieved perfect classification (AUROC 1.00; sensitivity 100%; specificity 100%), while sEV lipids still performed well (AUROC 0.76; sensitivity 80%; specificity 82%) (Figure 5G,J). Top contributing lipids included PA 36:2 and LPC 10:0 in sEVs and NAPE 30:11 and CDPDAG 20:2 in plasma, suggesting altered phospholipid metabolism and EV biogenesis (Figures S7 and S8, Table S7). Supporting AUPRC and calibration plots are shown in Figure S6B. Together, these data highlight the potential of lipid‐based biomarkers, particularly sEV lipids, to resolve diagnostic ambiguity between benign, borderline and low‐grade tumours where CA‐125 is uninformative.

**FIGURE 6:**
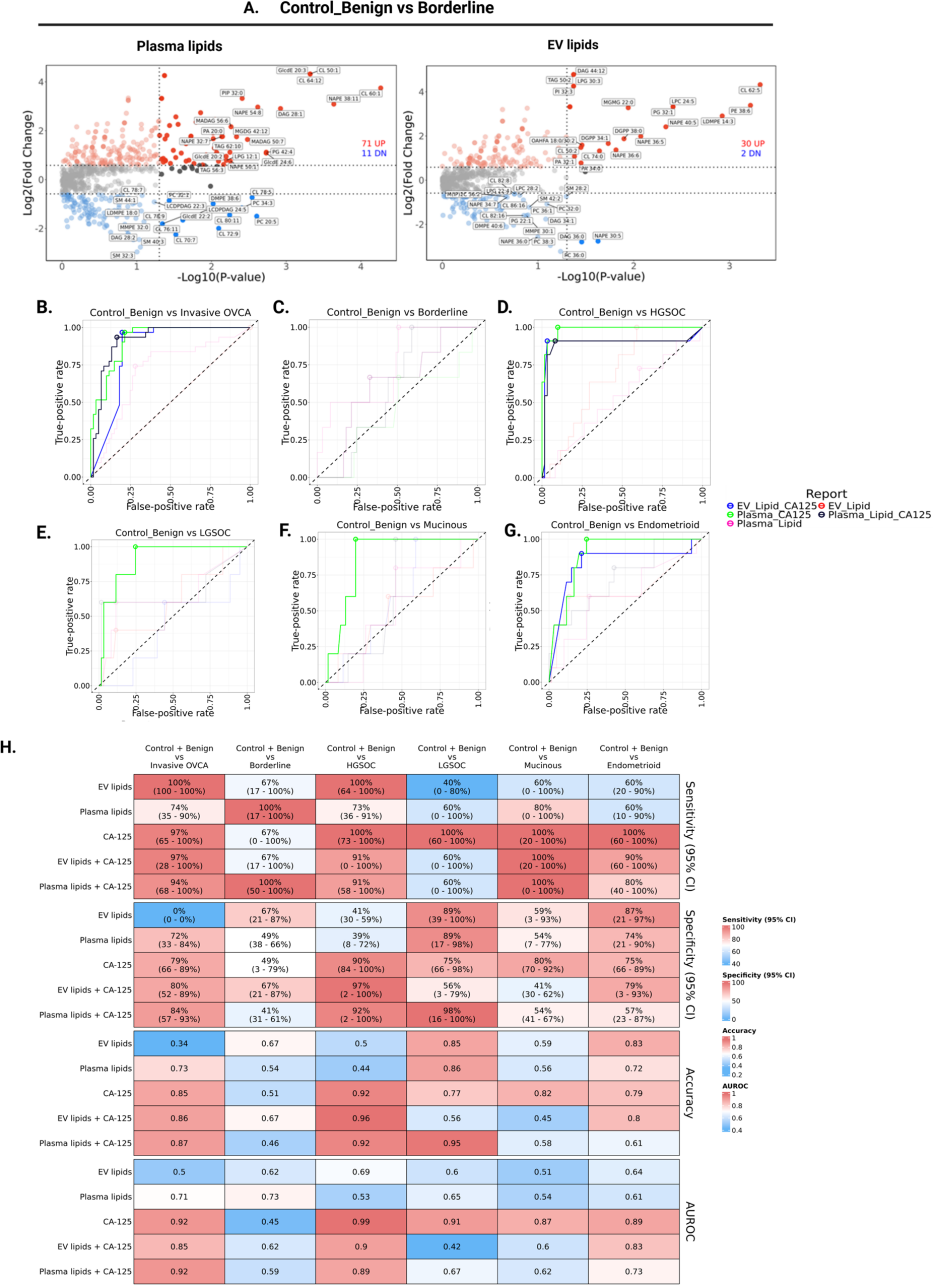
Differential enrichment and diagnostic performance of biomarker combinations distinguishing Ovarian cancer patients with non‐malignant samples. Volcano plot showing differential expression of plasma and sEV lipids in (A) Control + Benign versus Borderline. Overlaid Receiver Operating characteristics (ROC) curve for the classification performance of biomarker combinations EV lipids, plasma lipids, CA‐125, EV lipids + CA‐125 and plasma + CA‐125 for (B) Control+ Benign versus Invasive OVCA, (C) Control+ Benign versus Borderline, (D) Control + Benign versus HGSOC, (E) Control+ Benign versus LGSOC, (F) Control + Benign versus Mucinous and (G) Control + Benign versus Endometrioid carcinoma. (H) Table summarizing sensitivity and specificity (95% CI), accuracy and AUROC for each combination. All model performance metrics shown in this figure are derived from the independent test cohort (30% of samples), which was not used for model training or tuning (training cohort = 70% of samples).

To reduce variability among non‐cancer samples and improve model robustness, we combined healthy controls and benign cases into a single Control/Benign group. This group was then compared against ovarian cancer subtypes and borderline tumours using volcano plots (Figure 6A), ROC analyses (Figure 6B–G) and supplementary plots (Figure S11) For Control/Benign versus overall ovarian cancer, CA‐125 retained strong performance (AUROC 0.92; Figure 6H). However, for Control/Benign versus borderline, CA‐125 performed poorly (AUROC 0.45; sensitivity 67%; specificity 49%; Figure 6C,H). Plasma lipids improved sensitivity (AUROC 0.73; sensitivity 100%; specificity 49%), while sEV lipids provided a more balanced profile (AUROC 0.62; sensitivity 67%; specificity 67%) (Figure 6C,H). Key lipids distinguishing Control/Benign from borderline included LPS 16:1 and LPG 10:3 in sEVs and PC 34:3 and GlcdE 24:6 in plasma (Figures S7 and S8, Table S7). AUPRC and calibration curves for these comparisons are presented in Figure S6C. In summary, models based on CA‐125 alone, plasma lipids, sEV lipids and combined CA‐125 plus lipid panels showed that, for several key contrasts, control versus invasive OVCA, control versus borderline, control versus benign, benign versus borderline, benign versus LGSOC, Control/Benign versus invasive OVCA and Control/Benign versus borderline, adding plasma and/or sEV lipids to CA‐125 increased AUROC and/or AUPRC. In comparisons where CA‐125 already performed strongly, combined models were similar to CA‐125 alone.

**FIGURE 7:**
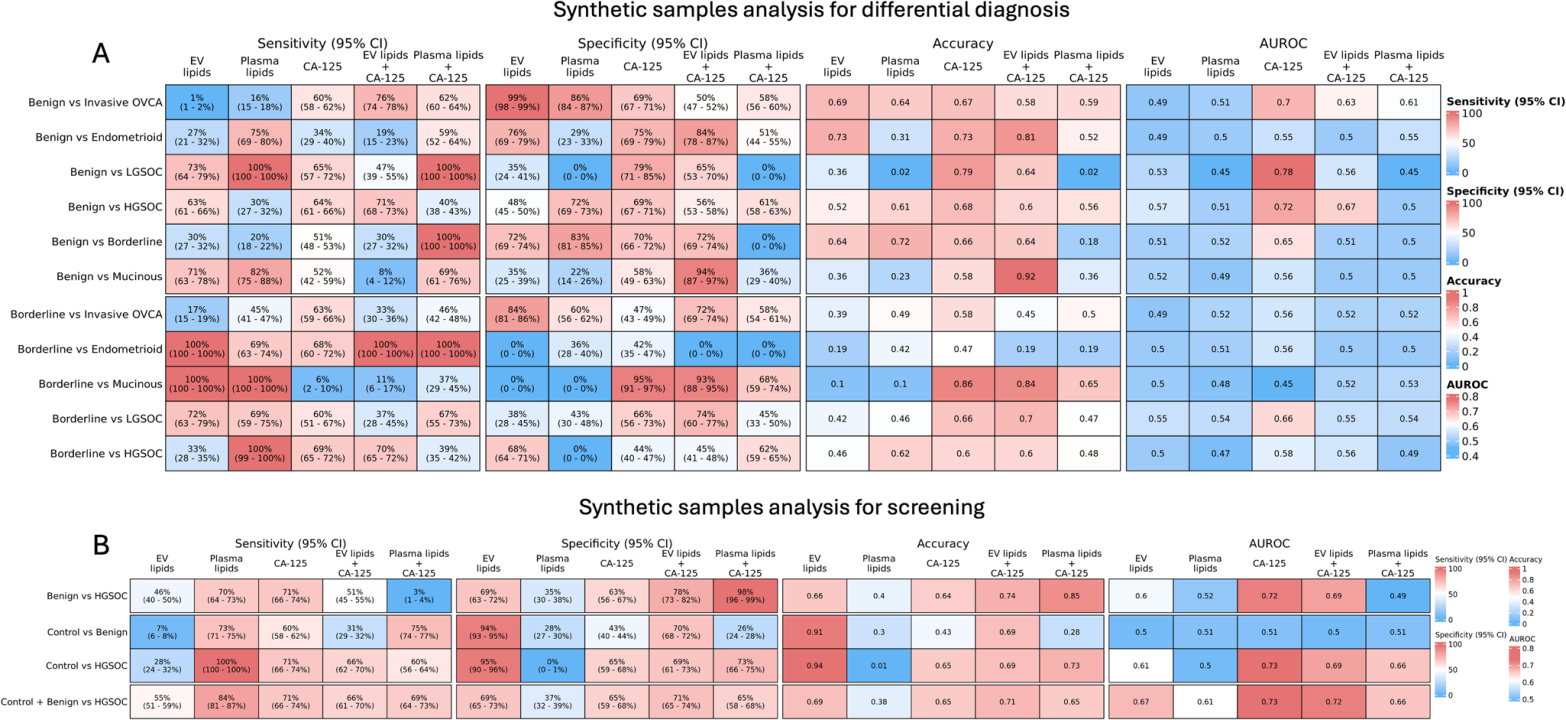
Generative AI‐based simulation of biomarker performance for differential diagnosis and screening in synthetic cohorts. Heatmaps displaying the classification performance of sEV lipids, plasma lipids, CA‐125 and their combinations (EV lipids + CA‐125; plasma lipids + CA‐125) across pairwise diagnostic comparisons in two synthetic cohorts generated using a Gaussian Copula Synthesizer. (A) Differential diagnosis scenario (*n* = 10,000 synthetic samples) evaluating the ability of each biomarker panel to distinguish between benign, borderline and malignant ovarian cancer subtypes, including invasive ovarian cancer (OVCA), endometrioid carcinoma, low‐grade serous ovarian cancer (LGSOC), high‐grade serous ovarian cancer (HGSOC) and mucinous carcinoma. (B) Screening scenario (*n* = 100,000 synthetic samples) evaluating discrimination between controls, benign adnexal masses and HGSOC. For each comparison, sensitivity (95% CI), specificity (95% CI), accuracy and AUROC are reported. Colour intensity reflects the magnitude of each metric, with deep red indicating high values and deep blue indicating low values, as shown in the colour scale bars on the right.

We further explored how biomarker panels would perform in larger, more representative populations by implementing a generative AI‐based modelling framework across two clinical scenarios: differential diagnosis (*n* = 10,000 synthetic samples) and screening (*n* = 100,000 synthetic samples) (see Section 2.12) (Figure 7). For differential diagnosis, sEV lipids combined with CA‐125 provided a notable advantage for specific histotype comparisons, achieving the highest accuracy for distinguishing benign from mucinous ovarian cancer (accuracy 0.92; specificity 94%), substantially outperforming CA‐125 alone (accuracy 0.58; specificity 58%) and plasma lipids (specificity 22%). Additionally, sEV lipids alone achieved 100% sensitivity for distinguishing borderline from both endometrioid and mucinous ovarian cancer, suggesting strong potential for identifying these subtypes (Figure 7A). For screening, sEV lipids alone demonstrated the strongest overall performance, achieving high accuracy for distinguishing controls from benign adnexal masses (accuracy 0.91; specificity 94%) and controls from high‐grade serous ovarian cancer (accuracy 0.94; specificity 95%) (Figure 7B). These exploratory simulations suggest that sEV lipids offer a diagnostic advantage for specific histotype discrimination and population‐level screening, but because synthetic cohorts are derived from the original limited dataset, these estimates should be interpreted with caution and confirmed in independent real‐world prospective cohorts.

### 3.4 Lipid‐Based Classification in Patients With Normal CA‐125

**FIGURE 8:**
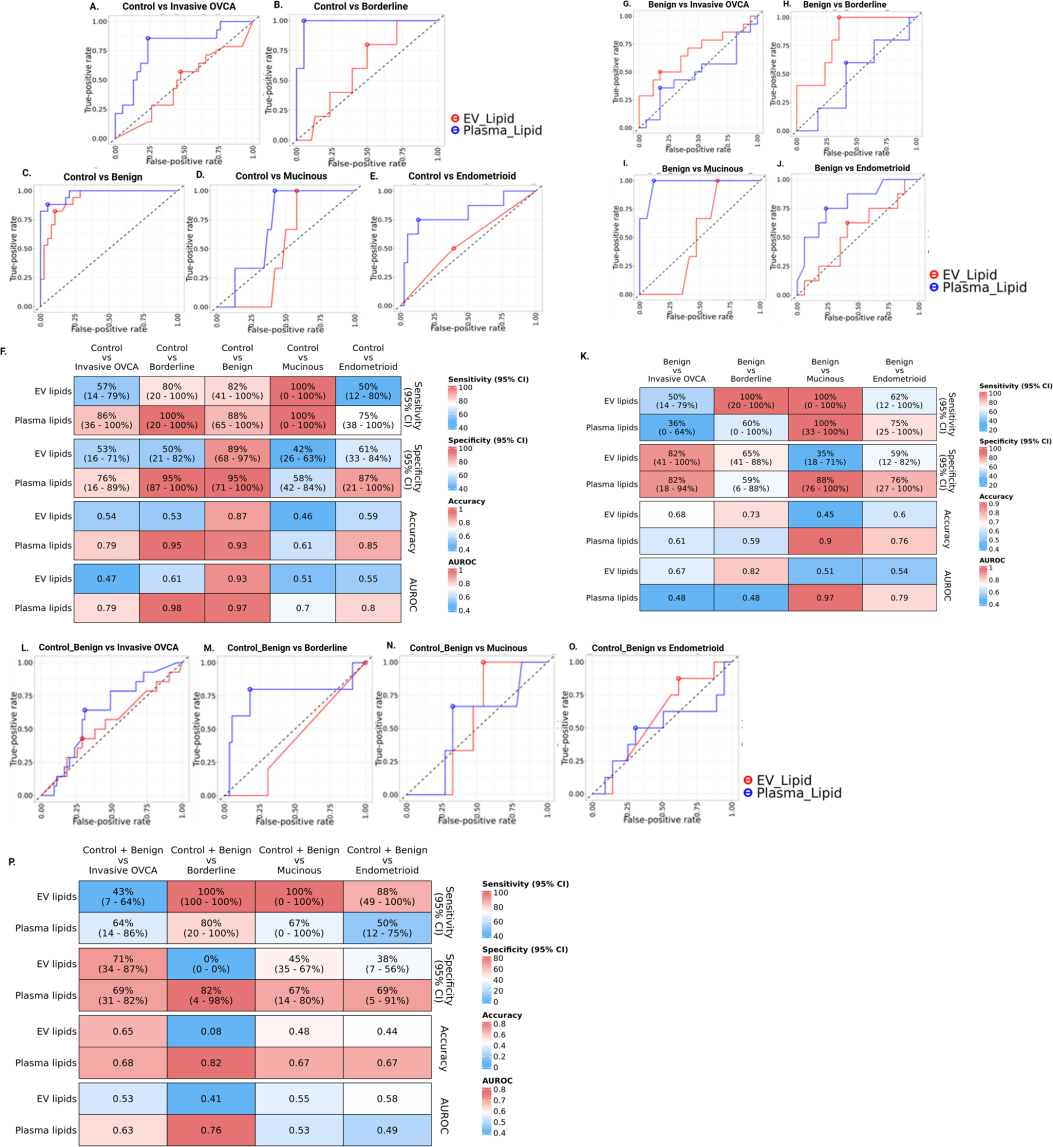
ROC curve analysis of biomarker performance in Ovarian cancer patients with CA‐125 levels <35 U/mL compared to controls. ROC curve for the classification performance of biomarker utilizing sEV lipids, plasma lipids in patients below CA‐125 cutoff. (A–E) Control versus Invasive OVCA, Control versus Borderline, Control versus Benign, Control versus Mucinous and Control versus Endometrioid carcinoma. (G–J) Benign versus Invasive OVCA, Benign versus Borderline, Benign versus Mucinous and Benign versus Endometrioid carcinoma. (L–O) Control + Benign versus Invasive OVCA, Control + Benign versus Borderline, Control + Benign versus Mucinous and Control + Benign versus Endometrioid carcinoma. (F, K, P) Table summarizing sensitivity and specificity (95% CI), accuracy and AUROC for respective combination. All model performance metrics shown in this figure are derived from the independent test cohort (30% of samples), which was not used for model training or tuning (training cohort = 70% of samples).

To assess diagnostic potential in patients with CA‐125 below the clinical threshold of 35 U/mL, we evaluated plasma and sEV lipid panels across multiple subtypes and comparisons (Figure 3A). Within this CA‐125‐negative subset, plasma lipids outperformed sEV lipids for distinguishing controls from invasive ovarian cancer, borderline tumours, benign disease, mucinous carcinoma and endometrioid carcinoma, with AUROCs of 0.79, 0.98, 0.97, 0.70 and 0.80, respectively (Figure 8A–E). Sensitivity, specificity and accuracy are summarised in Figure 8F. The top five contributing lipids (SHAP analysis) for control versus OVCA with CA‐125<35 U/mL are shown in Figure S12 and Table 2. For benign versus borderline and benign versus invasive OVCA, sEV lipids performed better than plasma lipids, achieving AUROCs of 0.82 and 0.67, respectively (Figure 8G,H). In the benign versus borderline comparison, key sEV lipids were LDMPE 14:3 and DGPP 38:0, whereas plasma lipids were NAPE 32:4 and CL 50:2. For benign versus invasive OVCA, discriminating sEV lipids included GlcdE 24:4 and GlcdE 22:3, and the leading plasma lipids were LCDPDAG 24:5 and PC 34:1 (Figure S12, Table 2). Plasma lipids showed particularly strong performance for benign versus mucinous carcinoma (AUROC 0.97; sensitivity 100%; specificity 88%), while benign versus endometrioid carcinoma showed more moderate discrimination (Figure 8I–K, Figure S12, Table 2). When control and benign samples were combined into a single non‐cancer reference group, comparisons with ovarian cancer cases yielded moderate classification performance (Figure 8L–P). Additional analyses comparing borderline tumours with invasive OVCA, mucinous and endometrioid carcinomas showed that sEV lipids achieved AUROCs of 0.80, 0.80 and 0.61, respectively (Figure S14D,E). Key sEV lipids included NAPE 30:5 and DAG 36:2 for borderline versus invasive OVCA, NAPE 36:0 and LPS 16:1 for borderline versus mucinous, and MGMG 18:0 and PG 20:4 for borderline versus endometrioid (Figure S12, Table 2). AUPRC and calibration plots for all CA‐125‐negative comparisons are shown in Figure S13. Overall, these findings demonstrate that both plasma and sEV lipid biomarkers provide superior diagnostic accuracy to CA‐125 in CA‐125‐negative patients. Plasma lipids achieved excellent performance for identifying borderline and benign conditions (AUROC 0.98 and 0.97; sensitivity 100% and 88%; specificity 95% for both). Plasma lipids also outperformed sEV lipids for invasive ovarian cancer (AUROC 0.79 vs. 0.47; sensitivity 86% vs. 57%; specificity 76% vs. 53%), mucinous carcinoma (AUROC 0.70 vs. 0.51; sensitivity 100% for both; specificity 58% vs. 42%) and endometrioid carcinoma (AUROC 0.80 vs. 0.55; sensitivity 75% vs. 50%; specificity 87% vs. 61%) (Figure 8A–F). Conversely, sEV lipids outperformed plasma for benign versus borderline (AUROC 0.82 vs. 0.48), benign versus invasive OVCA (0.67 vs. 0.48), borderline versus invasive OVCA (0.80 vs. 0.56) and borderline versus mucinous (0.80 vs. 0.73) (Figure 8G,H,K; Figure S14D,E). Collectively, these results underscore the value of lipid biomarkers, particularly sEV lipids, as complementary tools to CA‐125 for diagnostically challenging subtypes where CA‐125 alone is inadequate.

**TABLE 2:** Diagnostic performance and SHAP lipid features of EV and plasma lipid models in patients below the CA‐125 clinical cutoff (35U/mL).

	Sensitivity (95% CI)		Specificity (95% CI)		Accuracy		AUROC		AUPRC		Brier Score		Top_5_ SHAP	
Analysis	EV	Plasma	EV	Plasma	EV	Plasma	EV	Plasma	EV	Plasma	EV	Plasma	EV	Plasma
Control_vs_Benign	82% (41%–100%)	88% (65%–100%)	89% (68%–97%)	95% (71%–100%)	0.87	0.93	0.93	0.97	0.83	0.96	0.16	0.07	CL 76:2, LPS 30:0, CL 78:10, PA 36:2, PA 28:0	LPG 10:1, GlcdE 26:5, MADAG 56:9, NAPE 48:3, GlcdE 20:5
Control_vs_Borderline	80% (20%–100%)	100% (20%–100%)	50% (21%–82%)	95% (87%–100%)	0.53	0.95	0.61	0.98	0.13	0.86	0.19	0.07	CL 80:2, CL 74:0, LPG 22:0, PA 30:0, CL 76:8	NAPE 32:10, PG 42:4, NAPE 38:11, DAG 36:0, NAPE 32:4
Control_vs_Mucinous_carcinoma	100% (0%–100%)	100% (0%–100%)	42% (26%–63%)	58% (42%–84%)	0.46	0.61	0.51	0.7	0.95	0.11	0.08	0.08	LDMPE 12:2, LPS 18:2, LPG 10:4, CL 74:9, DGMG 20:4	GlcdE 22:3, LPS 20:2, NAPE 52:1, NAPE 38:2, OAHFA 16:0/24:1
Control_vs_Endometrioid_carcinoma	50% (12%–80%)	75% (38%–100%)	61% (33%–84%)	87% (21%–100%)	0.59	0.85	0.55	0.8	0.35	0.48	0.41	0.12	PC 34:2, MADAG 36:1, CL 66:8, TAG 60:8, PC 30:0	CL 70:8, DAG 36:0, MADAG 54:6, LPIP2 22:6, GlcdE 26:6
Control_vs_Invasive OVCA	57% (14%–79%)	86% (36%–100%)	53% (16%–71%)	76% (16%–89%)	0.54	0.79	0.47	0.79	0.72	0.61	0.29	0.16	NAPE 32:0, PG 24:1, CL 78:6, CL 80:2, SM 48:0	MADAG 54:6, LCDPDAG 20:2, PIP3 42:4, LPG 10:1, CL 80:11
Benign_vs_Borderline	100% (20%–100%)	60% (0%–100%)	65% (41%–88%)	59% (6%–88%)	0.73	0.59	0.82	0.48	0.64	0.78	0.23	0.3	LDMPE 14:3, DGPP 38:0, TAG 40:2, LPS 16:1, DAG 30:3	NAPE 32:4, CL 50:2, CL 56:6, TAG 56:5, CL 60:1
Benign_vs_Mucinous_carcinoma	100% (0%–100%)	100% (33%–100%)	35% (18%–71%)	88% (76%–100%)	0.45	0.90	0.51	0.97	0.14	0.99	0.17	0.16	MAG 26:2, GM3 34:1, M(IP)2C 36:2, PE 40:2, CL 40:4	GlcdE 22:6, PG 36:5, TAG 42:1, CL 82:0, DAG 30:2
Benign_vs_Endometrioid_carcinoma	62% (12%–100%)	75% (25%–100%)	59% (12%–82%)	76% (27%–100%)	0.60	0.76	0.54	0.79	0.33	0.67	0.23	0.18	DGPP 28:0, MADAG 40:1, PA 32:0, CDPDAG 20:1, CDPDAG 20:2	NAPE 48:3, TAG 40:0, CDPDAG 20:2, CDPDAG 20:3, CDPDAG 20:5
Benign_vs_Invasive OVCA	50% (14%–79%)	36% (0%–64%)	82% (41%–100%)	82% (18%–94%)	0.68	0.61	0.67	0.48	0.7	0.51	0.22	0.24	GlcdE 24:4, GlcdE 22:3, CL 54:2, MAG 26:4, GlcdE 26:3	LCDPDAG 24:5, PC 34:1, PS 42:3, CL 58:6, LPS 16:3
Borderline_vs_Mucinous_carcinoma	100% (0%–100%)	100% (0%–100%)	60% (20%–100%)	60% (20%–100%)	0.75	0.75	0.8	0.73	0.89	0.86	0.29	0.31	NAPE 36:0, LPS 16:1, PG 20:4, LPA 24:2, LPC 12:4	CL 60:4, OAHFA 16:0/32:2, PA 20:0, PC 38:6, CL 58:0
Borderline_vs_Endometrioid_carcinoma	25% (0%–75%)	75% (0%–100%)	100% (40%–100%)	60% (0%–100%)	0.54	0.69	0.61	0.58	0.62	0.41	0.41	0.25	MGMG 18:0, PG 20:4, DGPP 24:1, NAPE 32:0, CL 74:0	NAPE 38:11, CL 50:1, DGDG 30:2, NAPE 36:10, DGDG 32:5
Borderline_vs_Invasive OVCA	93% (7%–100%)	43% (21%–71%)	80% (20%–100%)	100% (0%–100%)	0.89	0.58	0.8	0.56	0.88	0.84	0.08	0.22	NAPE 30:5, DAG 36:2, CDPDAG 20:1, CDPDAG 20:2, CDPDAG 20:5	DMPE 38:6, NAPE 38:11, TAG 56:5, OAHFA 18:1/34:2, CL 54:0
Control_Benign_vs_Borderline	100% (100%–100%)	80% (20%–100%)	0% (0%–0%)	82% (4%–98%)	0.08	0.82	0.41	0.76	0.06	0.27	0.35	0.09	MMPE 32:1, MADAG 40:1, LPG 16:4, LPE 10:3, CL 76:6	CL 78:5, NAPE 32:4, PG 42:4, CL 84:11, LPG 18:2
Control_Benign_vs_Mucinous_carcinoma	100% (0%–100%)	67% (0%–100%)	45% (35%–67%)	67% (14%–80%)	0.48	0.67	0.55	0.53	0.05	0.05	0.19	0.05	MAG 26:2, DAG 30:3, LPE 18:2, DAG 36:3, GlcdE 24:2	NAPE 38:2, GlcdE 22:3, CL 64:12, MADAG 56:8, DAG 42:1
Control_Benign_vs_Endometrioid_carcinoma	88% (49%–100%)	50% (12%–75%)	38% (7%–56%)	69% (5%–91%)	0.44	0.67	0.58	0.49	0.14	0.86	0.15	0.16	CL 82:6, DAG 32:1, MAG 26:3, LPE 12:3, PG 32:0	LPG 14:0, CL 58:1, GlcdE 20:2, MADAG 52:6, CL 80:6
Control_Benign_vs_Invasive OVCA	43% (7%–64%)	64% (14%–86%)	71% (34%–87%)	69% (31%–82%)	0.65	0.68	0.53	0.63	0.8	0.25	0.34	0.21	NAPE 32:0, MAG 26:4, NAPE 32:8, LDMPE 10:3, GlcdE 26:3	NAPE 52:4, PC 34:1, MADAG 54:6, CL 84:13, CL 66:0

**FIGURE 9:**
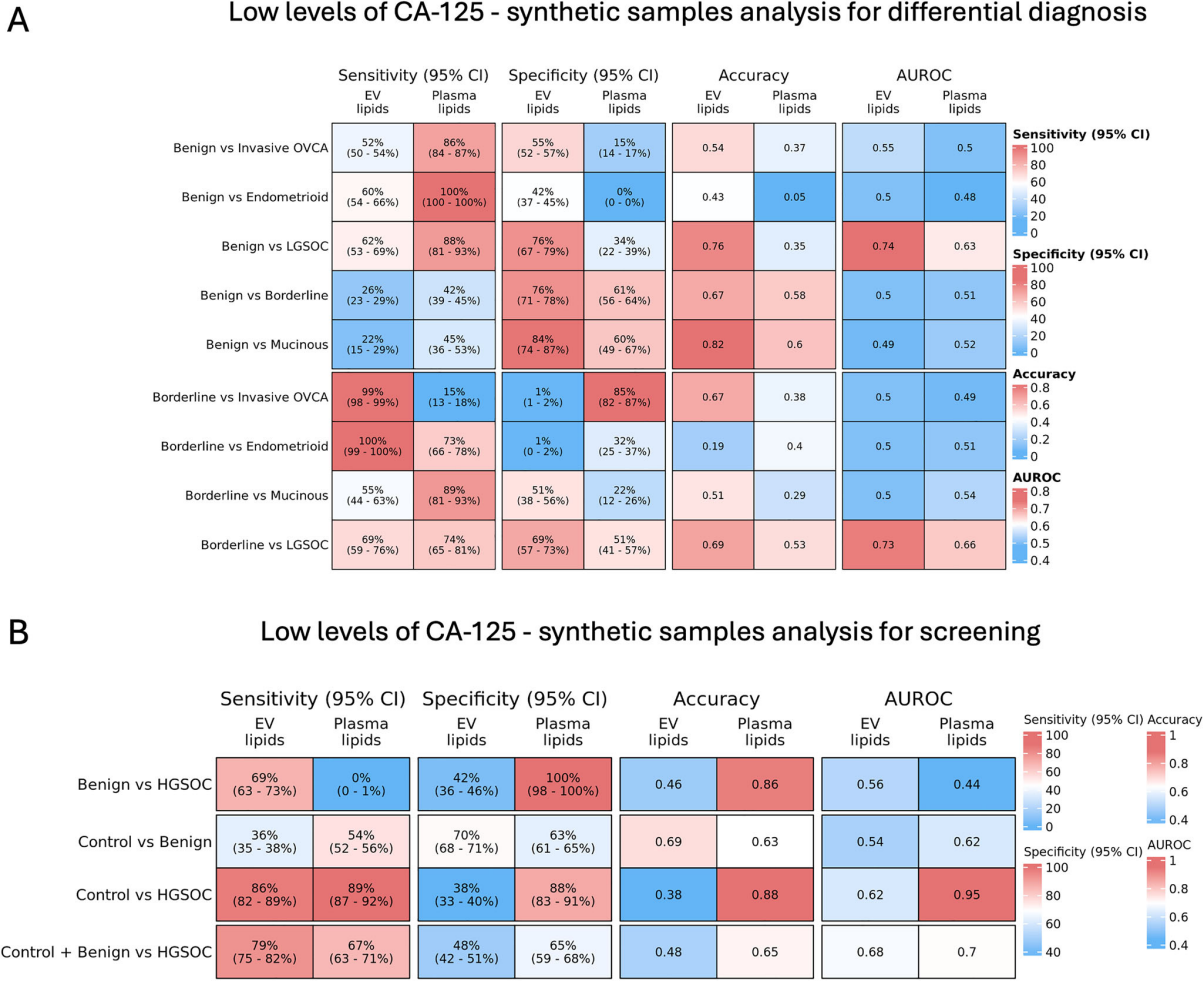
Generative AI‐based simulation of biomarker performance in patients with normal CA‐125 levels. Heatmaps displaying the classification performance of sEV lipids and plasma lipids across pairwise diagnostic comparisons in two synthetic cohorts generated using a Gaussian Copula Synthesizer, restricted to patients with normal CA‐125 levels to simulate clinical scenarios where standard CA‐125 triage would fail to identify malignancy. (A) Differential diagnosis scenario (*n* = 10,000 synthetic samples) evaluating the ability of each biomarker panel to distinguish between benign, borderline and malignant ovarian cancer subtypes—including invasive ovarian cancer (OVCA), endometrioid carcinoma, low‐grade serous ovarian cancer (LGSOC) and mucinous carcinoma—in women presenting with adnexal masses and normal CA‐125 levels. (B) Screening scenario (*n* = 100,000 synthetic samples) evaluating discrimination between controls, benign adnexal masses and high‐grade serous ovarian cancer (HGSOC) in a CA‐125‐negative population. For each comparison, sensitivity (95% CI), specificity (95% CI), accuracy and AUROC are reported. Colour intensity reflects the magnitude of each metric, with deep red indicating high values and deep blue indicating low values, as shown in the colour scale bars on the right.

In the synthetic differential diagnosis cohort (*n* = 10,000), generated from patients presenting with adnexal masses and normal CA‐125 levels to mimic a real‐world differential diagnosis scenario, EV lipids outperformed plasma lipids for distinguishing benign and borderline tumours from low‐grade serous ovarian cancer (LGSOC), achieving the highest AUROC values across both biomarker panels (benign vs. LGSOC: AUROC 0.74, sensitivity 62%, specificity 76% and borderline vs. LGSOC: AUROC 0.73, sensitivity 69%, specificity 69%) (Figure 9A). Importantly, these results demonstrate the potential of sEV lipids to add diagnostic value in the clinically challenging scenario where CA‐125 alone fails, leaving patients with malignancy but normal CA‐125 levels without a timely diagnosis of ovarian cancer. sEV lipids also demonstrated high specificity for benign versus mucinous carcinoma (84%) and benign versus borderline tumours (76%), with a notably high AUPRC for the latter comparison (0.81), reflecting strong discriminative performance in this imbalanced setting. For the screening scenario, a synthetic cohort of 100,000 women with normal CA‐125 levels was simulated to assess biomarker performance in a population where standard triage would fail to identify malignancy (Figure 9B). sEV lipids demonstrated moderate discriminative ability for distinguishing controls from all ovarian cancer cases (AUROC 0.62; sensitivity 86% [82%–89%]), and for distinguishing the combined group of controls and benign cases from ovarian cancer (AUROC 0.68; sensitivity 79% [75%–82%]), suggesting reasonable sensitivity for cancer detection in CA‐125‐negative women. Interestingly, plasma lipids outperformed sEV lipids for distinguishing controls from all ovarian cancer cases, achieving the highest performance across both panels (AUROC 0.95; sensitivity 89% [87%–92%]; specificity 88% [83%–91%]) and performed comparably to sEV lipids for the combined controls and benign versus ovarian cancer comparison (AUROC 0.70 vs. 0.68). Importantly, synthetic data quality was further supported by close agreement between real and synthetic marginal distributions and strong overlap in UMAP projections across both sEV lipid and plasma lipid cohorts for differential diagnosis and screening scenarios (Figure S15).

## 4 Discussion

Despite advances in surgical and systemic therapies, ovarian cancer remains the most lethal gynaecological malignancy, largely because most patients are diagnosed at an advanced stage when curative treatment is rarely achievable (Siegel et al. 2020, Matulonis et al. 2016). Central to this diagnostic failure is the continued reliance on CA‐125, a biomarker with well‐documented limitations: its sensitivity is particularly poor for borderline, benign and non‐high‐grade serous ovarian cancer subtypes, a pattern also evident in our cohort (Buas et al. 2021, Moss et al. 2005). Mechanistically, CA‐125 shedding into the bloodstream requires substantial disruption of tumour architecture (Kenemans et al. 1993), which may explain why over 50% of early‐stage cases present with normal CA‐125 levels (Zurawski et al. 1988, Nagele et al. 1995), precisely the population where early intervention would be most impactful. Compounding this, CA‐125 is elevated across a broad range of non‐malignant conditions, including inflammatory and benign gynaecological diseases, driving unacceptably high false‐positive rates (Vuento et al. 1997; Guppy and Rustin 2002). Together, these limitations create a critical diagnostic gap, particularly for the differential diagnosis of adnexal masses and the identification of histological subtypes that CA‐125 systematically fails to detect. Here, we address this gap by profiling sEV lipids as a complementary biomarker source, demonstrating their diagnostic advantage across multiple ovarian cancer subtypes, including in patients with normal CA‐125 levels, where conventional biomarkers fall short.

In this study we demonstrate that lipid‐based biomarkers improve diagnostic performance beyond CA‐125 in clinically challenging settings. CA‐125 retained strong discrimination for malignancy (AUROC 0.92–0.99for control/benign vs. invasive OVCA and HGSOC) but performed poorly for benign, borderline, mucinous and endometrioid tumours and in CA‐125‐negative patients (AUROC often <0.7; sensitivity ∼50%–60%). In contrast, plasma and sEV lipid panels achieved AUROC ≥0.9 with sensitivity and specificity ≥85%–90% for several key comparisons (control vs. benign/borderline; benign vs. LGSOC), and sEV lipids outperformed CA‐125 for benign versus borderline and low‐grade histotypes. The added value of sEV lipids was most evident in these differential‐diagnosis settings; plasma lipids contributed most strongly in case–control comparisons. Importantly, in the CA‐125<35 U/mL subgroup, lipid signatures, alone or combined with CA‐125, retained measurable discriminatory power, supporting their potential to fill the current diagnostic gap in early‐stage and CA‐125‐negative presentations. CA‐125 is not used as a stand‐alone test in clinical practice but interpreted together with symptoms, imaging and histopathology. Lipid‐based markers are therefore proposed as complementary tools within this multimodal pathway rather than as replacements.

Lipid metabolism is increasingly recognised as a hallmark of cancer, with tumour cells exhibiting extensive metabolic reprogramming to support proliferation, membrane synthesis and signalling (Broadfield et al. 2021). Several studies suggest that lipid alterations may precede clinical detection of ovarian cancer (Buas et al. 2021, Sah et al. 2024, Rong et al. 2025, Hada et al. 2019), positioning lipidomics as a promising avenue for biomarker discovery. However, despite growing evidence of lipid dysregulation across cancers (Zhao et al. 2019, Tania et al. 2010), lipid‐based biomarkers have not yet been integrated into clinical workflows. Plasma lipidomic studies in ovarian cancer have identified subtype‐ and stage‐specific alterations, including enrichment of lysophospholipids in early disease and broader lipid depletion in advanced stages (Rani et al. 2023). Similar trends have been reported in other malignancies, such as pancreatic and breast cancer (Wolrab et al. 2022, Chen et al. 2016), highlighting both shared and lipid remodelling processes. A key challenge in plasma lipidomics is the lack of disease specificity, as similar lipid alterations are observed in metabolic conditions such as cardiovascular disease, obesity and NAFLD (Stegemann et al. 2014, Yin et al. 2020, He et al. 2021). To address this, we incorporated analysis of sEVs, which are thought to carry tumour‐enriched molecular cargoes. Consistent with previous studies, we observed distinct lipid signatures in EVs, including enrichment of sphingomyelins, ceramides and phospholipids associated with cancer progression (Brzozowski et al. 2018, Skotland, Ekroos, et al. 2017, Hosseini‐Beheshti et al. 2012, Smolarz et al. 2021, Skotland, Sandvig, et al. 2017). These findings support the concept that EVs may provide a more tumour‐relevant lipid signal compared to bulk plasma.

Several recent lipidomics studies support the potential of circulating lipids as complementary biomarkers for ovarian cancer, but each has important differences relative to our study. Yagi et al. used plasma phospholipid species ratios to distinguish ovarian cancer from benign masses and healthy controls, reporting promising diagnostic performance (AUROC 0.84–0.95; sensitivity 90%–95%; specificity 70%–91%). However, their performance was not evaluated against CA‐125 in CA‐125 negative patients, limiting its applicability to this clinically challenging subgroup (Yagi et al. 2020). Buas et al. quantified >500 plasma lipids across two independent cohorts of women with malignant or benign adnexal masses and showed that adding specific lipid DAG (16:1/18:1) species to CA‐125 improved the accuracy of distinguishing early‐stage ovarian cancer cases from benign controls, increasing specificity by 15%–20% at 90% sensitivity in multivariate models. They also found that certain lipids were more altered in early‐stage disease, particularly DAGs, whereas lysophospholipids showed greater changes in later‐stage disease. However, the work was restricted to total plasma lipids and did not assess EV‐associated lipids or histotype‐specific performance (Buas et al. 2021).

Sah et al. performed untargeted serum lipidomics in a large cohort of Korean women with ovarian cancer and other gynaecological malignancies identifying broad reductions in most of the lipid classes but increases in some ceramides, triacylglycerols and diglycerides across disease spectrum. A 17‐lipid panel test set consisted mainly of ceramides, ether‐linked glycerophospholipids and gangliosides, discriminated ovarian cancer from nonovarian cancer cases with AUC of 0.85, sensitivity and specificity of 0.75 and 0.82, respectively. In particular, early‐stage ovarian cancer versus benign disease analysis yielded an AUC of 0.86, sensitivity of 0.81 and specificity 0.69. However, comparison against normal controls was not evaluated, and CA‐125 and HE4 were not incorporated. Borderline or low‐grade tumours were not specifically assessed (Sah et al., 2024). Giles et al. extended this work to two large symptomatic cohorts and, using multi‐omic machine learning approach, reported AUCs of 0.92 for ovarian cancer versus controls and 0.88 for early‐stage disease versus controls in the independent test cohort. Their proof‐of‐concept model included <20 serum lipid and protein features, including CA‐125, HE4, FRα and MUC1, but their design focused on triage in symptomatic women rather than for detailed CA‐125–negative and histotype‐specific stratification (Giles et al. 2025). In contrast, our study integrates untargeted lipidomics of both plasma and sEVs with CA‐125 across a well‐annotated cohort spanning benign, borderline, LGSOC, HGSOC, mucinous and endometrioid carcinomas, explicitly quantifies performance in CA‐125–negative patients, and directly compares single‐analyte and multimodal models using AUROC, AUPRC, calibration and power analyses. By dissecting plasma versus sEV lipid contributions, reporting histotype‐specific metrics, and testing models in a synthetic patient population, we provide a more detailed assessment where lipid biomarkers add value beyond CA‐125 and define specific scenarios, such as CA‐125–negative, benign versus borderline and benign versus LGSOC, for future clinical translation.

Our study extends previous work by applying untargeted lipidomics across multiple ovarian cancer subtypes, including cases with CA‐125 levels below the diagnostic threshold. Importantly, we demonstrate that lipid‐based models, particularly those derived from sEVs‐provide complementary diagnostic value in clinically challenging scenarios. Specific lipid species, including phosphatidic acids, lysophospholipids and glycosylated lipids, emerged as key contributors to classification models, consistent with their known roles in tumour signalling, membrane dynamics and metabolic reprogramming (Ghossoub et al. 2014, Baietti et al. 2012, Egea‐Jimenez and Zimmermann 2018; Chae et al. 2008). These findings highlight the biological relevance of lipid alterations in ovarian cancer and their potential utility as biomarkers. To further assess the robustness and scalability of our biomarker models, we implemented a generative artificial intelligence‐based modelling framework to simulate performance under expanded cohort conditions, as previously described (Joglekar et al. 2025). Using this approach, we simulated two distinct clinical scenarios: a differential diagnosis cohort (*n* = 10,000) and a population‐level screening cohort (*n* = 100,000), while preserving the underlying statistical properties of the original dataset. Across both scenarios, sEV lipids provided a diagnostic advantage for specific histotype comparisons and demonstrated meaningful performance in CA‐125‐negative patients. However, because the synthetic cohorts are derived from the original limited dataset, they cannot introduce biological or clinical variability absent from the source data, may overestimate model robustness, and cannot substitute for validation in independent prospective real‐world cohorts.

Despite these promising findings, several limitations should be considered. First, reproducibility remains a challenge in lipidomics, particularly across different extraction and analytical platforms. Variability in lipid extraction efficiency, ionisation and identification may impact the consistency of detected lipid species, highlighting the need for standardised workflows. Second, we observed variability in diagnostic performance between plasma and sEV‐derived lipids across comparisons. While sEV lipids often provided improved specificity and subtype discrimination, plasma lipids performed better in certain contexts, suggesting that these compartments capture complementary but distinct biological information. Third, the most informative lipid species differed between classification tasks, reflecting underlying biological heterogeneity but also limiting the identification of a single, universally applicable biomarker panel. Additionally, potential co‐isolation of lipoproteins and other contaminants during sEV enrichment may influence lipid profiles, particularly for species such as cardiolipins (Paradies et al. 2019, Deguchi et al. 2000). Although such lipids may also have functional roles in EV biology (Haraszti et al. 2016), their interpretation requires caution. Our unsupervised analyses confirmed clear separation between plasma and sEV lipidomes, supporting their distinct biological origins, but future studies integrating lipoprotein profiling and high‐purity sEV isolation methods will be essential to fully disentangle these signals (Ter‐Ovanesyan et al. 2023).

From a clinical perspective, lipid‐based biomarkers demonstrated improved diagnostic performance compared to CA‐125 in several key scenarios, particularly for benign, borderline and low‐grade tumours, and in CA‐125 negative patients. However, these biomarkers are not intended to replace existing diagnostic approaches. Rather, they may provide complementary information within the current clinical workflow, which includes imaging and histopathological confirmation. Age‐related effects on lipid profiles were considered and an age‐stratified sensitivity analysis confirmed that lipid signatures retained comparable discrimination after age adjustment, supporting the robustness of the findings to differences in age distribution between groups.

Finally, our study has several limitations related to cohort composition and generalisability. First, the small sample size of several diagnostic subgroups, which directly drives the reduced statistical power observed for these comparisons. A further limitation concerns our use of a generative AI–based modelling framework to simulate a larger patient cohort. The Gaussian Copula Synthesizer reproduces the multivariate statistical structure of the input data. However, the synthetic cohort was used only for exploratory performance evaluation and was not used to train the classifiers. Finally, the absence of ovarian clear cell carcinoma reflects local case availability and represents an important gap that should be addressed in future studies.

In conclusion, this study demonstrates that lipid‐based biomarkers, particularly those derived from sEVs, have the potential to improve the detection and classification of ovarian cancer across subtypes, including in patients with normal CA‐125 levels. While challenges remain in standardisation, reproducibility and validation, these findings support the integration of lipidomics into multimodal diagnostic strategies aimed at improving early detection and patient outcomes. This study was conducted within an ISO17025‐aligned Quality Management System (QMS), which standardised sample handling, EV isolation, lipid extraction and analytical workflows and supports reproducibility and future clinical translation of lipid‐based biomarkers.

## Author Contributions

**Shikha Rani**: conceptualization, formal analysis, investigation, writing – original draft, writing – review and editing. **Dominic Guanzon**: formal analysis, methodology, visualization. **Andrew Lai**: formal analysis, methodology, visualization. **Katherin Scholz‐Romero**: methodology, investigation. **Lewis Perrin**: formal analysis, investigation, methodology. **Rohan Lourie**: methodology, investigation. **Vaibhavi Joshi**: methodology, investigation. **Amy McCart Reed**: methodology, investigation. **Kaltin Ferguson**: investigation, methodology. **Aase Handberg**: supervision, methodology, investigation, writing – review and editing. **Andreas Möller**: writing – review and editing, investigation, methodology, supervision. **Carlos Salomon**: investigation, methodology, supervision, writing – review and editing, conceptualization, funding acquisition, project administration, writing – original draft.

## Funding

C.S. is supported by The Medical Research Future Fund (MRF1199984), National Health and Medical Research Council (NHMRC 1195451), The Donald & Joan Wilson Foundation Ltd. (2020000323) and the Ovarian Cancer Research Foundation (OCRF, 2018001167). A.M. was supported by the Chinese University of Hong Kong (IDBF23MED14), the Innovation and Technology Commission, Hong Kong SAR (PiH/048‐050/22GS), the Global STEM scheme (GSP153), the Hong Kong Jockey Club Charities Trust (2023‐0031) and Cancer Australia by grant 2010799 awarded through the 2021 Priority‐driven Collaborative Cancer Research Scheme.

## Ethics Statement

This study was performed in accordance with the Declaration of Helsinki. The overall analysis was approved by the Ethics committee of the University of Queensland and Mater Hospital (2023/HE002226), Human Research Ethics Committees at The University of Queensland (2005000785) and Royal Brisbane and Women's Hospital (RBWH 2005/022).

## Conflicts of Interest

The authors declare no conflicts of interest.

## Supporting information




**File S1**: LC‐MS negative and positive ion total ion chromatograms(TIC) for lipid extracts from BIOIVT gender pooled A)plasma and B)plasma derived small EVs using lipid extraction methods ‐BD, BUME, Folch, MTBE, SPE_cartridge and SPE_plate.

**File S2-S3**: Principal component Analysis(PCA) of lipid composition in (A) plasma and (B) EV samples (n=3) from LLE and SPE extraction methods. Concentration and mode particle size of sEVs across patient groups.

**File S4**: Volcano plot analysis of differential lipid abundance in plasma and EVs across ovarian cancer groups, compared to control.

**File S5**: Global structure and heatmap of lipid profiles.

**File S6**: Area under the precision‐recall curve (AUPRC) and Calibration curve for ovarian cancer patients comparing with Control.

**File S7**: SHAP (SHapley Additive exPlanations) analysis identifying top predictive features contributing to EV lipids biomarker model performance distinguishing Control or benign vs Ovarian cancer patients.

**File S8**: SHAP (SHapley Additive exPlanations) analysis identifying top predictive features contributing to plasma lipids biomarker model performance distinguishing Control or benign vs Ovarian cancer patients.

**File S9**: Confusion matrices for lipid and CA‐125 models.

**File S10**: Volcano plot analysis of differential lipid abundance in plasma and EVs across ovarian cancer groups, compared to Benign.

**File S11**: Volcano plot analysis of differential lipid abundance in plasma and EVs across ovarian cancer groups, compared to Benign.

**File S12**: SHAP (SHapley Additive exPlanations) analysis identifying top predictive features contributing to plasma lipids and EV lipids biomarker model performance in Ovarian cancer patients with CA125 levels <35 U/mL.

**File S13**: Area under the precision‐recall curve (AUPRC) and Calibration curve for ovarian cancer patients with CA125 levels <35U/mL.

**File S14**: A) Overlaid ROC curve analysis and performance of biomarker combinations comparing Borderline patients with Ovarian cancer patients.

**File S15**: Quality assessment of synthetic cohorts generated for differential diagnosis and screening simulations.

**Supporting Information**: jev270348‐sup‐0015‐SuppMat.docx

**Supporting Information**: jev270348‐sup‐17‐Figures‐legend.docx

**Supporting Information**: jev270348‐sup‐18‐Table‐S1‐S6‐SR.docx

**Supporting Information**: jev270348‐sup‐0016‐Table‐S7.xlsx

## Data Availability

All data supporting the findings of this study are available within the article and the supplemental information, or from the corresponding author upon reasonable request.
