# Supplementary material for: Small Extracellular Vesicle and Total Lipid Profiles Provide a Diagnostic Advantage Over CA‐125 Across Ovarian Cancer Subtypes
Source: J Extracell Vesicles. 2026 Sep 1;15(9):e70348. doi: 10.1002/jev2.70348 (PMC13533800; doi:10.1002/jev2.70348)
Supplement: Supplementary file 1 [file JEV2-15-e70348-s004.docx]

**Table S1: List of lipid classes selected for analysis in LipidView software (SCIEX)**

| **Lipid Categories** | | | | | |
| --- | --- | --- | --- | --- | --- |
| **Lipid Classes** | **Glycerophospholipids** | **Glycosphingolipids** | **Glycerolipids** | **Sterol Lipids** | **Fatty Acyls** |
|  | Phosphatidic Acid (PA) | Sphingomyelin (SM) | Diacylglyceride (DAG) | Cholesteryl Ester (CE) | Free Fatty Acid (FFA) |
|  | Phosphatidylcholine (PC) | Ceramide (Cer) | Triacylglyceride (TAG) | Desmoestryl ester (Des) | Methyl Ester (ME) |
|  | Phosphatidylethanolamine (PE) | Mannosyl-inositolPceramide (MIPC) | Monoacylglyceride (MAG) | Ergosteryl Ester (Erg) | Ethyl Ester (EE) |
|  | Phosphatidylglycerol (PG) | Mannosyl-dinositolPceramide (M1P2C) | Monoalkyl DAG (MADAG) | Lanosteryl Ester (Lan) | Glycidol Ester (GlcdE) |
|  | Phosphatidylinositol (PI) | Dihexosylceramide (Hex2Cer) | Monogalactosyl MAG (MGMG) | Stigmasteryl Ester (Sti) | O-16:0-Hydroxy Fatty Acid (OAHFA, 16:0/) |
|  | Phosphatidylserine (PS) | Trihexosylceramide (Hex3Cer) | Digalactosyl MAG (DGMG) |  | O-16:1-Hydroxy Fatty Acid (OAHFA, 16:1/) |
|  | Lyso-Phosphatidic Acid (LPA) | Monosialoganglioside (GM3) | Monogalactosyl DAG (MGDG) |  | O-18:0-Hydroxy Fatty Acid (OAHFA, 18:0/) |
|  | Lyso-Phosphatidylcholine (LPC) | Monosialoganglioside GalNAc (GM2) | Digalactosyl DAG (DGDG) |  | O-18:1-Hydroxy Fatty Acid (OAHFA, 18:1/) |
|  | Lyso-Phosphatidylethanolamine (LPE) | Monosialoganglioside GalGalNAc (GM1) | Sulphoquinovosyl DAG (SQDG) |  | O-18:2-Hydroxy Fatty Acid (OAHFA, 18:2/) |
|  | Lyso-Phosphatidylglycerol (LPG) | Disialoganglioside (GD3) |  |  |  |
|  | Lyso-Phosphatidylinositol (LPI) | Disialoganglioside GalNAc (GD2) |  |  |  |
|  | Lyso-Phosphatidylserine (LPS) | Disialoganglioside GalGalNAc (GD1) |  |  |  |
|  | Cardiolipin (CL) | Trisialoganglioside (GT3) |  |  |  |
|  | DG Pyrophosphate (DGPP) | Trisialoganglioside GalNAc (GT2) |  |  |  |
|  | Monomethyl PE (MMPE) | Trisialoganglioside GalGalNAc (GT1) |  |  |  |
|  | Dimethyl PE (DMPE) | Long Chain Base (LCB) |  |  |  |
|  | PI Phosphate (PIP) | Ceramide Cilatine (CerCl) |  |  |  |
|  | PI Biphosphate (PIP2) | Sulphatides (sGalCer) |  |  |  |
|  | PI Triphosphate (PIP3) | Ceramide -phosphate (CerP) |  |  |  |
|  | Cytidine Diphosphate DAG (CDPDAG) | Ceramide phosphoethanolamine (CerPE) |  |  |  |
|  | N-acyl PE (NAPE) | Inositolphosphhorylceramides (IPC) |  |  |  |

Table S1: Lipid species were annotated post-acquisition using LipidView software (SCIEX). The table lists lipid classes grouped into glycerophospholipids, glycosphingolipids, glycerolipids, sterol lipids and fatty acyls.

**Table S2: Synthetic data quality metrics for EV and plasma cohorts in ovarian cancer differential diagnosis**

| **Property** | **Score** | **Analysis** |
| --- | --- | --- |
| Column Shapes | 0.224404 | EV Control vs OVCA subtypes |
| Column Pair Trends | 0.973333 | EV Control vs OVCA subtypes |
| Overall | 0.598868 | EV Control vs OVCA subtype |
| Column Shapes |  | Plasma Control vs OVCA subtypes |
| Column Pair Trends | 0.5 | Plasma Control vs OVCA subtypes |
| Overall | 0.5 | Plasma Control vs OVCA subtypes |

Table S2: Quality of synthetic data was evaluated using the evaluate_quality function from the synthetic data vault (SDV) framework, which compares the statistical properties of synthetic data against original dataset. Validation included assessment of the overall quality score (global similarity, 0-1 scale), column shapes (similarity of marginal distributions of individual variables), and the column pair trends (preservation of pairwise correlations). Scores are shown for EV and plasma cohorts, each comparing control samples against ovarian cancer subtypes in the simulated differential diagnosis cohort. The higher scores indicate better preservation of the original data structure.

**Table S3A: Performance of 14 machine-learning classifiers across biomarker panels and comparisons.**

|  | **Model** | **Accuracy** | **AUC** | **Recall** | **Prec**. | **F1** | **Kappa** | **MCC** | **Specificity** | **TT (Sec)** |
| --- | --- | --- | --- | --- | --- | --- | --- | --- | --- | --- |
| **EV lipids_CA125** | | | | | | | | | | |
| Benign_vs_Borderline_Model_metrics | Ada Boost Classifier | 0.9333 | 0.55 | 0.6 | 0.55 | 0.5667 |  | 0.55 | 0.9167 | 0.122 |
| Benign_vs_Endometrioid_carcinoma_Model_metrics | Gradient Boosting Classifier | 0.7333 | 0.7333 | 0.5 | 0.4 | 0.4333 | 0.3133 | 0.3333 | 0.8333 | 0.155 |
| Benign_vs_HGSOC_Model_metrics | Ada Boost Classifier | 0.825 | 0.9667 | 0.75 | 0.65 | 0.6667 | 0.59 | 0.6232 | 0.8833 | 0.136 |
| Benign_vs_LGSOC_Model_metrics | Random Forest Classifier | 0.8333 | 0.475 | 0 | 0 | 0 |  | 0 | 1 | 0.137 |
| Benign_vs_Mucinous_carcinoma_ Model_metrics | Extra Trees Classifier | 0.8333 | 0.5 | 0 | 0 | 0 |  | 0 | 1 | 0.127 |
| Benign_vs_OVCA_all_Model_metrics | Random Forest Classifier | 0.6367 | 0.7618 | 0.7333 | 0.635 | 0.6471 | 0.2589 | 0.2798 | 0.5167 | 0.207 |
| Borderline_vs_Endometrioid_carcinoma_ Model_metrics | Decision Tree Classifier | 0.8 | 0.5 | 0.9 | 0.8 | 0.8333 |  | 0.4 | 0.4 | 0.115 |
| Borderline_vs_HGSOC_Model_metrics | Ada Boost Classifier | 0.85 | 0.45 | 0.9 | 0.8 | 0.8333 |  | 0.3 | 0.4 | 0.136 |
| Borderline_vs_LGSOC_Model_metrics | Logistic Regression | 0.75 | 0.6667 | 0.3333 | 0.3333 | 0.3333 |  | 0.3333 | 1 | 0.6117 |
| Borderline_vs_Mucinous_carcinoma_Model_metrics | Gradient Boosting Classifier | 0.6667 | 0.8333 | 0.3333 | 0.25 | 0.2778 |  | 0.1667 | 0.8333 | 0.1217 |
| Borderline_vs_OVCA_all_Model_metrics | Gradient Boosting Classifier | 0.8917 | 0.55 | 0.9417 | 0.95 | 0.9371 |  | 0.4 | 0.4 | 0.168 |
| Control_Benign_vs_Borderline_Model_metrics | Ada Boost Classifier | 0.9143 | 0.5 | 0 | 0 | 0 |  | 0 | 1 | 0.183 |
| Control_Benign_vs_Endometrioid_ Model_metrics | Ada Boost Classifier | 0.8304 | 0.8333 | 0.3 | 0.25 | 0.2667 | 0.202 | 0.2054 | 0.9167 | 0.141 |
| Control_Benign_vs_HGSOC_Model_metrics | Gradient Boosting Classifier | 0.9429 | 0.9333 | 0.9 | 0.75 | 0.8 | 0.7765 | 0.7936 | 0.95 | 0.207 |
| Control_Benign_vs_LGSOC_Model_metrics | Extra Trees Classifier | 0.9286 | 0.3667 | 0 | 0 | 0 |  | 0 | 1 | 0.133 |
| Control_Benign_vs_Mucinous_carcinoma_Model_metrics | Extreme Gradient Boosting | 0.8952 | 0.3167 | 0 | 0 | 0 |  | 0 | 0.9667 | 0.31 |
| Control_Benign_vs_OVCA_all_Model_metrics | Ada Boost Classifier | 0.76 | 0.8875 | 0.625 | 0.6867 | 0.5967 | 0.4461 | 0.4873 | 0.8333 | 0.147 |
| Control_vs_Benign_Model_metrics | Random Forest Classifier | 0.8667 | 0.9556 | 0.75 | 0.8667 | 0.78 | 0.7048 | 0.7157 | 0.9417 | 0.144 |
| Control_vs_Borderline_Model_metrics | Ada Boost Classifier | 0.81 | 0.475 | 0.2 | 0.1 | 0.1333 |  | 0.0816 | 0.875 | 0.147 |
| Control_vs_Endometrioid_carcinoma_ Model_metrics | Extreme Gradient Boosting | 0.85 | 0.85 | 0.5 | 0.45 | 0.4667 | 0.4212 | 0.4279 | 0.9417 | 0.283 |
| Control_vs_HGSOC_Model_metrics | Ada Boost Classifier | 0.96 | 0.9375 | 0.9 | 0.85 | 0.8667 | 0.8545 | 0.8612 | 0.975 | 0.163 |
| Control_vs_LGSOC_Model_metrics | Extreme Gradient Boosting | 0.895 | 0.425 | 0.1 | 0.05 | 0.0667 |  | 0.0612 | 0.975 | 0.282 |
| Control_vs_Mucinous_carcinoma_ Model_metrics | Extreme Gradient Boosting | 0.87 | 0.4417 | 0 | 0 | 0 |  | -0.0333 | 0.9667 | 0.262 |
| Control_vs_OVCA_all_ Model_metrics | Ada Boost Classifier | 0.9 | 0.95 | 0.9 | 0.8917 | 0.8838 | 0.7968 | 0.8127 | 0.9 | 0.137 |
| **EV lipids** | | | | | | | | | | |
| Benign_vs_Borderline_Model_metrics | Ada Boost Classifier | 0.9333 | 0.55 | 0.6 | 0.55 | 0.5667 |  | 0.55 | 0.9167 | 0.191 |
| Benign_vs_Endometrioid_carcinoma_ Model_metrics | Logistic Regression | 0.6917 | 0.65 | 0.1 | 0.1 | 0.1 | 0.0667 | 0.0667 | 0.9667 | 0.457 |
| Benign_vs_HGSOC_Model_metrics | Extreme Gradient Boosting | 0.8583 | 0.8667 | 0.8 | 0.7 | 0.7333 | 0.68 | 0.7 | 0.9 | 0.274 |
| Benign_vs_LGSOC_Model_metrics | Extra Trees Classifier | 0.8667 | 0.475 | 0.1 | 0.1 | 0.1 |  | 0.1 | 1 | 0.163 |
| Benign_vs_Mucinous_carcinoma_ Model_metrics | Extra Trees Classifier | 0.8333 | 0.475 | 0 | 0 | 0 |  | 0 | 1 | 0.136 |
| Benign_vs_OVCA_all_Model_metrics | Extra Trees Classifier | 0.6033 | 0.7833 | 0.8333 | 0.5667 | 0.6702 | 0.1182 | 0.1245 | 0.2833 | 0.183 |
| Borderline_vs_Endometrioid_carcinoma_ Model_metrics | Decision Tree Classifier | 0.8 | 0.5 | 0.8 | 0.75 | 0.7667 |  | 0.4 | 0.5 | 0.113 |
| Borderline_vs_HGSOC_Model_metrics | Gradient Boosting Classifier | 0.95 | 0.55 | 1 | 0.95 | 0.9667 |  | 0.5 | 0.5 | 0.156 |
| Borderline_vs_LGSOC_Model_metrics | Logistic Regression | 0.75 | 0.6667 | 0.3333 | 0.3333 | 0.3333 |  | 0.3333 | 1 | 0.6167 |
| Borderline_vs_Mucinous_carcinoma_ Model_metrics | Quadratic Discriminant Analysis | 0.5 | 0.8333 | 0.3333 | 0.25 | 0.2778 | 0.1667 | 0.1667 | 0.6667 | 0.1183 |
| Borderline_vs_OVCA_all_Model_metrics | Gradient Boosting Classifier | 0.9167 | 0.55 | 0.9417 | 0.975 | 0.9514 |  | 0.5 | 0.5 | 0.172 |
| Control_Benign_vs_Borderline_Model_metrics | Ada Boost Classifier | 0.9143 | 0.4833 | 0 | 0 | 0 |  | 0 | 1 | 0.143 |
| Control_Benign_vs_Endometrioid_Model_metrics | Linear Discriminant Analysis | 0.7286 | 0.5833 | 0.6 | 0.3167 | 0.4 | 0.2756 | 0.2888 | 0.75 | 0.251 |
| Control_Benign_vs_HGSOC_Model_metrics | Extreme Gradient Boosting | 0.8482 | 0.6679 | 0 | 0 | 0 | 0 | 0 | 1 | 0.274 |
| Control_Benign_vs_LGSOC_Model_metrics | Extra Trees Classifier | 0.9286 | 0.3417 | 0 | 0 | 0 |  | 0 | 1 | 0.144 |
| Control_Benign_vs_Mucinous_carcinoma_Model_metrics | Extra Trees Classifier | 0.9286 | 0.3333 | 0 | 0 | 0 |  | 0 | 1 | 0.132 |
| Control_Benign_vs_OVCA_all_Model_metrics | Naive Bayes | 0.6856 | 0.5333 | 0.0667 | 0.2 | 0.1 | 0.08 | 0.1 | 1 | 0.117 |
| Control_vs_Benign_Model_metrics | Random Forest Classifier | 0.9 | 0.9556 | 0.8 | 0.8667 | 0.8133 | 0.781 | 0.7972 | 0.975 | 0.142 |
| Control_vs_Borderline_Model_metrics | Ada Boost Classifier | 0.81 | 0.475 | 0.2 | 0.1 | 0.1333 |  | 0.0816 | 0.875 | 0.132 |
| Control_vs_Endometrioid_carcinoma_Model_metrics | Ada Boost Classifier | 0.81 | 0.8667 | 0.4 | 0.4 | 0.4 | 0.3167 | 0.3167 | 0.9167 | 0.14 |
| Control_vs_HGSOC_Model_metrics | Logistic Regression | 0.76 | 0.6167 | 0 | 0 | 0 | -0.025 | -0.025 | 0.975 | 0.42 |
| Control_vs_LGSOC_Model_metrics | Extra Trees Classifier | 0.895 | 0.375 | 0 | 0 | 0 |  | 0 | 1 | 0.146 |
| Control_vs_Mucinous_carcinoma_ Model_metrics | Ada Boost Classifier | 0.77 | 0.35 | 0 | 0 | 0 |  | -0.0333 | 0.8667 | 0.147 |
| Control_vs_OVCA_all_Model_metrics | Extreme Gradient Boosting | 0.5857 | 0.6417 | 0.4583 | 0.4333 | 0.4336 | 0.1409 | 0.1472 | 0.6833 | 0.255 |
|  |  |  |  |  |  |  |  |  |  |  |
| **Plasma CA125** | | | | | | | | | | |
| Benign_vs_Borderline_Model_metrics | Logistic Regression | 0.45 | 0.35 | 0.2 | 0.15 | 0.1667 | -0.01 | 0 | 0.5167 | 0.435 |
| Benign_vs_Endometrioid_carcinoma_Model_metrics | Extra Trees Classifier | 0.85 | 0.95 | 0.8 | 0.65 | 0.7 | 0.63 | 0.6577 | 0.8667 | 0.037 |
| Benign_vs_HGSOC_Model_metrics | Logistic Regression | 0.9167 | 0.9667 | 0.9 | 0.8 | 0.8333 | 0.8 | 0.8155 | 0.9333 | 0.373 |
| Benign_vs_LGSOC_Model_metrics | Logistic Regression | 0.7333 | 0.45 | 0.3 | 0.25 | 0.2667 |  | 0.25 | 0.7833 | 0.483 |
| Benign_vs_Mucinous_carcinoma_Model_metrics | Logistic Regression | 0.7 | 0.5 | 0.2 | 0.15 | 0.1667 |  | 0.15 | 0.7833 | 0.543 |
| Benign_vs_OVCA_all_Model_metrics | Logistic Regression | 0.6633 | 0.9167 | 0.5167 | 0.7917 | 0.599 | 0.3696 | 0.4262 | 0.8833 | 0.386 |
| Borderline_vs_Endometrioid_carcinoma_Model_metrics | Logistic Regression | 0.65 | 0.5 | 0.6 | 0.55 | 0.5667 |  | 0.3 | 0.5 | 0.367 |
| Borderline_vs_HGSOC_Model_metrics | Logistic Regression | 0.95 | 0.6 | 0.9 | 0.9 | 0.9 |  | 0.5 | 0.6 | 0.38 |
| Borderline_vs_LGSOC_Model_metrics | Logistic Regression | 0.75 | 0.6667 | 0.5 | 0.5 | 0.5 |  | 0.3333 | 0.8333 | 0.735 |
| Borderline_vs_Mucinous_carcinoma_ Model_metrics | K Neighbors Classifier | 0.8333 | 0.6667 | 0.8333 | 0.6667 | 0.7222 |  | 0.5 | 0.6667 | 0.0083 |
| Borderline_vs_OVCA_all_Model_metrics | Logistic Regression | 0.6 | 0.5 | 0.55 | 0.85 | 0.6567 | 0.17 | 0.1911 | 0.5 | 0.532 |
| Control_Benign_vs_Borderline_ Model_metrics | Logistic Regression | 0.6357 | 0.4167 | 0.3 | 0.1333 | 0.1833 | 0.0865 | 0.0984 | 0.6548 | 7.415 |
| Control_Benign_vs_Endometrioid_ Model_metrics | Random Forest Classifier | 0.8179 | 0.9024 | 0.8 | 0.4583 | 0.5567 | 0.4629 | 0.5097 | 0.819 | 0.03 |
| Control_Benign_vs_HGSOC_Model_metrics | Logistic Regression | 0.9571 | 0.9833 | 0.9 | 0.8 | 0.8333 | 0.8176 | 0.8291 | 0.9667 | 0.365 |
| Control_Benign_vs_LGSOC_Model_metrics | Logistic Regression | 0.8738 | 0.5 | 0.4 | 0.3 | 0.3333 |  | 0.3291 | 0.8833 | 0.871 |
| Control_Benign_vs_Mucinous_carcinoma_Model_metrics | Logistic Regression | 0.7952 | 0.4667 | 0.3 | 0.25 | 0.2667 |  | 0.2312 | 0.8167 | 7.062 |
| Control_Benign_vs_OVCA_all_Model_metrics | Logistic Regression | 0.7922 | 0.9514 | 0.5667 | 0.7383 | 0.5951 | 0.4816 | 0.5188 | 0.9 | 0.473 |
| Control_vs_Benign_Model_metrics | Logistic Regression | 0.6571 | 0.6889 | 0.5333 | 0.5083 | 0.5038 | 0.246 | 0.2457 | 0.7167 | 0.468 |
| Control_vs_Borderline_Model_metrics | Logistic Regression | 0.675 | 0.375 | 0.3 | 0.1583 | 0.19 |  | 0.0667 | 0.6917 | 0.471 |
| Control_vs_Endometrioid_carcinoma_ Model_metrics | Logistic Regression | 0.85 | 0.9667 | 0.9 | 0.65 | 0.7333 | 0.6394 | 0.6729 | 0.8417 | 0.651 |
| Control_vs_HGSOC_Model_metrics | Logistic Regression | 0.98 | 1 | 0.9 | 0.9 | 0.9 | 0.9 | 0.9 | 1 | 0.361 |
| Control_vs_LGSOC_Model_metrics | Logistic Regression | 0.96 | 0.45 | 0.4 | 0.35 | 0.3667 |  | 0.3612 | 0.975 | 0.345 |
| Control_vs_Mucinous_carcinoma_ Model_metrics | Logistic Regression | 0.885 | 0.475 | 0.5 | 0.35 | 0.4 |  | 0.3802 | 0.8667 | 0.533 |
| Control_vs_OVCA_all_Model_metrics | Logistic Regression | 0.8143 | 0.975 | 0.6083 | 0.9667 | 0.7124 | 0.5983 | 0.6513 | 0.975 | 0.375 |
|  |  |  |  |  |  |  |  |  |  |  |
| **Plasma lipids CA125** | | | | | | | | | | |
| Benign_vs_Borderline_Model_metrics | Extra Trees Classifier | 0.8 | 0.325 | 0 | 0 | 0 |  | 0 | 1 | 0.278 |
| Benign_vs_Endometrioid_carcinoma_Model_metrics | Ada Boost Classifier | 0.8 | 0.8333 | 0.6 | 0.5333 | 0.55 | 0.4867 | 0.5 | 0.9 | 0.229 |
| Benign_vs_HGSOC_Model_metrics | Logistic Regression | 0.8 | 0.9167 | 0.4 | 0.4 | 0.4 | 0.4 | 0.4 | 1 | 0.623 |
| Benign_vs_LGSOC_Model_metrics | Decision Tree Classifier | 0.9333 | 0.45 | 0.4 | 0.4 | 0.4 |  | 0.4 | 0.9667 | 0.112 |
| Benign_vs_Mucinous_carcinoma__Model_metrics | Random Forest Classifier | 0.8333 | 0.4 | 0 | 0 | 0 |  | 0 | 1 | 0.137 |
| Benign_vs_OVCA_all_Model_metric | Extreme Gradient Boosting | 0.7733 | 0.8931 | 0.8083 | 0.8167 | 0.7852 | 0.5301 | 0.5732 | 0.7167 | 0.314 |
| Borderline_vs_Endometrioid_carcinoma_Model_metrics | Quadratic Discriminant Analysis | 0.6 | 0.5 | 0.9 | 0.6 | 0.7 |  | 0 | 0 | 0.108 |
| Borderline_vs_HGSOC_Model_metrics | Extra Trees Classifier | 0.85 | 0.6 | 1 | 0.85 | 0.9 |  | 0.3 | 0.3 | 0.161 |
| Borderline_vs_LGSOC_Model_metrics | Logistic Regression | 0.9167 | 0.6667 | 0.6667 | 0.6667 | 0.6667 |  | 0.6667 | 1 | 0.9333 |
| Borderline_vs_Mucinous_carcinoma_Model_metrics | Random Forest Classifier | 0.5 | 0.8333 | 0.3333 | 0.25 | 0.2778 | 0.1667 | 0.1667 | 0.6667 | 0.2033 |
| Borderline_vs_OVCA_all_Model_metrics | Logistic Regression | 0.8 | 0.5 | 0.95 | 0.85 | 0.881 |  | 0 | 0 | 0.63 |
| Control_Benign_vs_Borderline__Model_metrics | Random Forest Classifier | 0.9143 | 0.5667 | 0 | 0 | 0 |  | 0 | 1 | 0.302 |
| Control_Benign_vs_Endometrioid_carcinoma_Model_metrics | Extreme Gradient Boosting | 0.8589 | 0.8333 | 0.4 | 0.3 | 0.3333 | 0.2855 | 0.2967 | 0.9357 | 0.288 |
| Control_Benign_vs_HGSOC_Model_metrics | Gradient Boosting Classifier | 0.9304 | 0.9333 | 0.75 | 0.7 | 0.7 | 0.6776 | 0.6946 | 0.9667 | 0.253 |
| Control_Benign_vs_LGSOC_Model_metrics | Ada Boost Classifier | 0.9429 | 0.4833 | 0.2 | 0.2 | 0.2 |  | 0.1833 | 0.9833 | 0.163 |
| Control_Benign_vs_Mucinous_carcinoma_ Model_metrics | Random Forest Classifier | 0.9286 | 0.4333 | 0 | 0 | 0 |  | 0 | 1 | 0.192 |
| Control_Benign_vs_OVCA_all_Model_metrics | Ada Boost Classifier | 0.8167 | 0.9556 | 0.7583 | 0.7933 | 0.7164 | 0.5935 | 0.6387 | 0.85 | 0.258 |
| Control_vs_Benign_Model_metrics | Logistic Regression | 0.9524 | 0.9653 | 0.9167 | 0.975 | 0.9324 | 0.8958 | 0.909 | 0.975 | 0.45 |
| Control_vs_Borderline_Model_metrics | Logistic Regression | 0.895 | 0.6 | 0.1 | 0.1 | 0.1 |  | 0.1 | 1 | 0.429 |
| Control_vs_Endometrioid_carcinoma_Model_metrics | Extreme Gradient Boosting | 0.835 | 0.9 | 0.3 | 0.25 | 0.2667 | 0.2545 | 0.2612 | 0.975 | 0.292 |
| Control_vs_HGSOC_Model_metrics | Decision Tree Classifier | 0.96 | 0.9375 | 0.9 | 0.85 | 0.8667 | 0.8545 | 0.8612 | 0.975 | 0.161 |
| Control_vs_LGSOC_Model_metrics | Logistic Regression | 0.895 | 0.475 | 0 | 0 | 0 |  | 0 | 1 | 0.633 |
| Control_vs_Mucinous_carcinoma_Model_metrics | Random Forest Classifier | 0.895 | 0.45 | 0 | 0 | 0 |  | 0 | 1 | 0.192 |
| Control_vs_OVCA_all_Model_metrics | Ada Boost Classifier | 0.9143 | 1 | 0.9333 | 0.9 | 0.9029 | 0.8271 | 0.8461 | 0.9 | 0.249 |
|  |  |  |  |  |  |  |  |  |  |  |
| **Plasma lipids** | | | | | | | | | | |
| Benign_vs_Borderline_Model_metrics | Quadratic Discriminant Analysis | 0.8 | 0.35 | 0 | 0 | 0 |  | 0 | 1 | 0.211 |
| Benign_vs_Endometrioid_carcinoma_Model_metrics | Extreme Gradient Boosting | 0.7 | 0.7833 | 0.5 | 0.3333 | 0.3833 | 0.2667 | 0.3 | 0.8 | 0.515 |
| Benign_vs_HGSOC_Model_metrics | Logistic Regression | 0.8 | 0.9167 | 0.4 | 0.4 | 0.4 | 0.4 | 0.4 | 1 | 0.492 |
| Benign_vs_LGSOC_Model_metrics | Ada Boost Classifier | 1 | 0.5 | 0.5 | 0.5 | 0.5 |  | 0.5 | 1 | 0.167 |
| Benign_vs_Mucinous_carcinoma_Model_metrics | Random Forest Classifier | 0.8333 | 0.4 | 0 | 0 | 0 |  | 0 | 1 | 0.197 |
| Benign_vs_OVCA_all_Model_metrics | Extreme Gradient Boosting | 0.5933 | 0.7694 | 0.625 | 0.62 | 0.5867 | 0.154 | 0.1798 | 0.5167 | 0.273 |
| Borderline_vs_Endometrioid_carcinoma_Model_metrics | Logistic Regression | 0.6 | 0.4 | 0.9 | 0.6 | 0.7 |  | 0 | 0 | 0.69 |
| Borderline_vs_HGSOC_Model_metrics | Extra Trees Classifier | 0.8 | 0.5 | 1 | 0.8 | 0.8667 |  | 0.2 | 0.2 | 0.129 |
| Borderline_vs_LGSOC_Model_metrics | Logistic Regression | 0.9167 | 0.6667 | 0.6667 | 0.6667 | 0.6667 |  | 0.6667 | 1 | 8.5783 |
| Borderline_vs_Mucinous_carcinoma_ Model_metrics | Gradient Boosting Classifier | 0.6667 | 0.8333 | 0.3333 | 0.25 | 0.2778 |  | 0.1667 | 0.8333 | 0.1233 |
| Borderline_vs_OVCA_all_Model_metrics | Logistic Regression | 0.8 | 0.5 | 0.95 | 0.85 | 0.881 |  | 0 | 0 | 8.099 |
| Control_Benign_vs_Borderline_Model_metrics | Random Forest Classifier | 0.9143 | 0.5333 | 0 | 0 | 0 |  | 0 | 1 | 0.2 |
| Control_Benign_vs_Endometrioid_ carcinoma_Model_metrics | Ada Boost Classifier | 0.9 | 0.8333 | 0.3 | 0.3 | 0.3 | 0.3 | 0.3 | 1 | 0.163 |
| Control_Benign_vs_HGSOC_Model_metrics | Gradient Boosting Classifier | 0.7946 | 0.7738 | 0.55 | 0.4033 | 0.44 | 0.338 | 0.3577 | 0.8357 | 0.386 |
| Control_Benign_vs_LGSOC_Model_metrics | Ada Boost Classifier | 0.9429 | 0.4667 | 0.2 | 0.15 | 0.1667 |  | 0.1645 | 0.9833 | 0.193 |
| Control_Benign_vs_Mucinous_carcinoma_Model_metrics | Random Forest Classifier | 0.9286 | 0.375 | 0 | 0 | 0 |  | 0 | 1 | 0.254 |
| Control_Benign_vs_OVCA_all_Model_metrics | Random Forest Classifier | 0.6233 | 0.661 | 0.3 | 0.3417 | 0.2995 | 0.0779 | 0.081 | 0.7881 | 0.236 |
| Control_vs_Benign_Model_metrics | Logistic Regression | 0.9524 | 0.9653 | 0.9167 | 0.975 | 0.9324 | 0.8958 | 0.909 | 0.975 | 0.591 |
| Control_vs_Borderline_Model_metrics | Logistic Regression | 0.895 | 0.6 | 0.1 | 0.1 | 0.1 |  | 0.1 | 1 | 0.64 |
| Control_vs_Endometrioid_carcinoma_ Model_metrics | Logistic Regression | 0.85 | 0.8833 | 0.4 | 0.4 | 0.4 | 0.3667 | 0.3667 | 0.9667 | 0.443 |
| Control_vs_HGSOC_Model_metrics | Quadratic Discriminant Analysis | 0.78 | 0.7417 | 0 | 0 | 0 | 0 | 0 | 1 | 0.134 |
| Control_vs_LGSOC_Model_metrics | Logistic Regression | 0.87 | 0.475 | 0 | 0 | 0 |  | 0 | 0.975 | 0.652 |
| Control_vs_Mucinous_carcinoma_Model_metrics | Ada Boost Classifier | 0.845 | 0.4292 | 0 | 0 | 0 |  | 0 | 0.95 | 0.226 |
| Control_vs_OVCA_all_Model_metrics | Random Forest Classifier | 0.7286 | 0.8083 | 0.6417 | 0.7033 | 0.6521 | 0.4324 | 0.456 | 0.7833 | 0.161 |

|  | **Top model Selected** | | | | |
| --- | --- | --- | --- | --- | --- |
| **Analysis** | **EV lipids+CA125** | **EV lipids** | **Plasma CA125** | **Plasma lipids CA125** | **Plasma lipids** |
| **Control_vs_Benign** | Random Forest Classifier | Random Forest Classifier | Logistic Regression | Logistic Regression | Logistic Regression |
| **Control_vs_Borderline** | Ada Boost Classifier | Ada Boost Classifier | Logistic Regression | Logistic Regression | Logistic Regression |
| **Control_vs_HGSOC** | Ada Boost Classifier | Logistic Regression | Logistic Regression | Decision Tree Classifier | Quadratic Discriminant Analysis |
| **Control_vs_LGSOC** | Extreme Gradient Boosting | Extra Trees Classifier | Logistic Regression | Logistic Regression | Logistic Regression |
| **Control_vs_Mucinous_carcinoma** | Extreme Gradient Boosting | Ada Boost Classifier | Logistic Regression | Random Forest Classifier | Ada Boost Classifier |
| **Control_vs_Endometrioid_carcinoma** | Extreme Gradient Boosting | Ada Boost Classifier | Logistic Regression | Extreme Gradient Boosting | Logistic Regression |
| **Control_vs_Invasive OVCA** | Ada Boost Classifier | Extreme Gradient Boosting | Logistic Regression | Ada Boost Classifier | Random Forest Classifier |
| **Benign_vs_Borderline** | Ada Boost Classifier | Ada Boost Classifier | Logistic Regression | Extra Trees Classifier | Quadratic Discriminant Analysis |
| **Benign_vs_HGSOC** | Ada Boost Classifier | Extreme Gradient Boosting | Logistic Regression | Logistic Regression | Logistic Regression |
| **Benign_vs_LGSOC** | Random Forest Classifier | Extra Trees Classifier | Logistic Regression | Decision Tree Classifier | Ada Boost Classifier |
| **Benign_vs_Mucinous_carcinoma** | Extra Trees Classifier | Extra Trees Classifier | Logistic Regression | Random Forest Classifier | Random Forest Classifier |
| **Benign_vs_Endometrioid_carcinoma** | Gradient Boosting Classifier | Logistic Regression | Extra Trees Classifier | Ada Boost Classifier | Extreme Gradient Boosting |
| **Benign_vs_Invasive OVCA** | Random Forest Classifier | Extra Trees Classifier | Logistic Regression | Extreme Gradient Boosting | Extreme Gradient Boosting |
| **Borderline_vs_HGSOC** | Ada Boost Classifier | Gradient Boosting Classifier | Logistic Regression | Extra Trees Classifier | Extra Trees Classifier |
| **Borderline_vs_LGSOC** | Logistic Regression | Logistic Regression | Logistic Regression | Logistic Regression | Logistic Regression |
| **Borderline_vs_Mucinous_carcinoma** | Gradient Boosting Classifier | Quadratic Discriminant Analysis | K Neighbors Classifier | Random Forest Classifier | Gradient Boosting Classifier |
| **Borderline_vs_Endometrioid_carcinoma** | Decision Tree Classifier | Decision Tree Classifier | Logistic Regression | Quadratic Discriminant Analysis | Logistic Regression |
| **Borderline_vs_Invasive OVCA** | Gradient Boosting Classifier | Gradient Boosting Classifier | Logistic Regression | Logistic Regression | Logistic Regression |
| **Control_Benign_vs_Borderline** | Ada Boost Classifier | Ada Boost Classifier | Logistic Regression | Random Forest Classifier | Random Forest Classifier |
| **Control_Benign_vs_HGSOC** | Gradient Boosting Classifier | Extreme Gradient Boosting | Logistic Regression | Gradient Boosting Classifier | Gradient Boosting Classifier |
| **Control_Benign_vs_LGSOC** | Extra Trees Classifier | Extra Trees Classifier | Logistic Regression | Ada Boost Classifier | Ada Boost Classifier |
| **Control_Benign_vs_Mucinous_carcinoma** | Extreme Gradient Boosting | Extra Trees Classifier | Logistic Regression | Random Forest Classifier | Random Forest Classifier |
| **Control_Benign_vs_Endometrioid_carcinoma** | Ada Boost Classifier | Linear Discriminant Analysis | Random Forest Classifier | Extreme Gradient Boosting | Ada Boost Classifier |
| **Control_Benign_vs_Invasive OVCA** | Ada Boost Classifier | Naive Bayes | Logistic Regression | Ada Boost Classifier | Random Forest Classifier |

Table S3A: For each biomarker panel and comparison, 14 machine-learning classifiers were trained and evaluated in the independent test set. A single algorithm with highest area under the receiver operating characteristic curve (AUROC), together with favorable sensitivity, specificity and calibration, was selected for each panel and comparison. Performance metrics for all 14 classifiers are provided with AUROC, recall (sensitivity), precision, F1-score, Cohen’s kappa, Matthews correlation coefficient (MCC), specificity and training time (TT, seconds).

**Table S3B. Age comparison between diagnostic groups and estimated contribution of age to lipid biomarker performance.**

| **Comparison** | **Mean age, Group 1 (years)** | **Mean age, Group 2 (years)** | **Absolute age difference (years)** | **Age p-value (t-test)** | **Best lipid-signature AUROC** | **Estimated age contribution to biomarker performance** |
| --- | --- | --- | --- | --- | --- | --- |
| Control vs Benign | 53.0 | 52.3 | 0.7 | 0.9755 | **0.96** | **Very low (<5%)** |
| Control vs Borderline | 53.0 | 49.7 | 3.3 | 0.6440 | **1.00** | **Very low (<5%)** |
| Control vs HGSOC | 53.0 | 66.2 | 13.2 | 0.0065 | **1.00** | **Moderate (10–20%)** |
| Control vs LGSOC | 53.0 | 69.8 | 16.8 | 0.0117 | **0.96** | **Moderate (10–20%)** |
| Control vs Mucinous carcinoma | 53.0 | 45.0 | 8.0 | 0.2883 | **0.94** | **Low (5–10%)** |
| Control vs Endometrioid carcinoma | 53.0 | 52.7 | 0.3 | 0.9568 | **0.94** | **Very low (<5%)** |
| Control vs Invasive OVCA | 53.0 | 59.2 | 6.2 | 0.0647 | **0.96** | **Low (5–10%)** |
| Benign vs Borderline | 52.3 | 49.7 | 2.6 | 0.6743 | **0.74** | **Very low (<5%)** |
| Benign vs HGSOC | 52.3 | 66.2 | 13.9 | 0.0254 | **0.95** | **Moderate (10–20%)** |
| Benign vs LGSOC | 52.3 | 69.8 | 17.5 | 0.0312 | **1.00** | **Moderate (10–20%)** |
| Benign vs Mucinous carcinoma | 52.3 | 45.0 | 7.3 | 0.4725 | **0.75** | **Low (5–10%)** |
| Benign vs Endometrioid carcinoma | 52.3 | 52.7 | 0.4 | 0.9479 | **0.92** | **Very low (<5%)** |
| Benign vs Invasive OVCA | 52.3 | 59.2 | 6.9 | 0.1218 | **0.85** | **Low (5–10%)** |
| Borderline vs HGSOC | 49.7 | 66.2 | 16.5 | 0.0345 | **0.97** | **Moderate (10–20%)** |
| Borderline vs LGSOC | 49.7 | 69.8 | 20.1 | 0.0436 | **0.83** | **Moderate (10–20%)** |
| Borderline vs Mucinous carcinoma | 49.7 | 45.0 | 4.7 | 0.5209 | **0.75** | **Very low (<5%)** |
| Borderline vs Endometrioid carcinoma | 49.7 | 52.7 | 3.0 | 0.7681 | **0.80** | **Very low (<5%)** |
| Borderline vs Invasive OVCA | 49.7 | 59.2 | 9.5 | 0.1809 | **0.88** | **Low (5–10%)** |

Table S3B: Mean age (years) for each diagnostic group pair, absolute age difference, and t-test p-values are reported for all pairwise comparisons. The best lipid-signature AUROC represents the highest discriminative performance achieved across all biomarker panels for each comparison. The estimated age contribution to biomarker performance was derived from an age-stratified sensitivity analysis and reflects the approximate proportion of the observed AUROC attributable to age differences between groups, categorised as very low (<5%), low (5–10%), or moderate (10–20%). Comparisons with statistically significant age differences (p<0.05), notably those involving HGSOC and LGSOC, which tend to affect older women, showed moderate age contributions (10–20%); however, lipid signatures maintained strong discriminative performance across all comparisons (AUROC 0.74–1.00), supporting the independent diagnostic value of the lipid biomarkers beyond age effects.

**Table S4: Clinical characteristics of the ovarian cancer cohort (n = 30)**

| **Histology type** | **Tumour status** | **BMI (kg/m²)** | **Stage** |
| --- | --- | --- | --- |
| High-grade serous carcinoma | Malignant | 46.3 | IV |
| High-grade serous carcinoma | Malignant | 31.6 | IVB |
| High-grade serous carcinoma | Malignant | 15.9 | IVB |
| High-grade serous carcinoma | Malignant | 20.9 | III |
| High-grade serous carcinoma | Malignant | NA | NA |
| High-grade serous carcinoma | Malignant | 24.1 | IC3 |
| High-grade serous carcinoma | Malignant | 20.2 | IVB |
| High-grade serous carcinoma | Malignant | 21.4 | IIIC |
| High-grade serous carcinoma | Malignant | 23.6 | IIIA2 |
| High-grade serous carcinoma | Malignant | 34.3 | III |
| High-grade serous carcinoma | Malignant | NA | IVA |
| Low-grade serous carcinoma | Malignant | NA | IV |
| Low-grade serous carcinoma | Malignant | 22.3 | IIIC |
| Low-grade serous carcinoma | Malignant | NA | ≥III |
| Low-grade serous carcinoma | Malignant | 25.7 | IA |
| Low-grade serous carcinoma | Malignant | 29.5 | NA |
| Mucinous carcinoma | Malignant | 36.1 | IIIB |
| Mucinous carcinoma | Malignant | 30.5 | IA |
| Mucinous carcinoma | Malignant | 39.0 | IIIC |
| Mucinous carcinoma | Malignant | 38.9 | IC2 |
| Mucinous carcinoma | Malignant | 22.0 | IIIC |
| Endometrioid carcinoma | Malignant | NA | IC3 |
| Endometrioid carcinoma | Malignant | 24.1 | IV |
| Endometrioid carcinoma | Malignant | 59.9 | IC3 |
| Endometrioid carcinoma | Malignant | 49.0 | IVB |
| Endometrioid carcinoma | Malignant | 17.8 | IA |
| Endometrioid carcinoma | Malignant | 34.0 | IA |
| Endometrioid carcinoma | Malignant | 60.0 | IC1 |
| Endometrioid carcinoma | Malignant | 38.0 | IVB |
| Endometrioid carcinoma | Malignant | 40.0 | IA |

Table S4: Tumour stage was classified according to the FIGO (International Federation of Gynecology and Obstetrics) staging system. Briefly, Stage I disease is confined to the ovaries or fallopian tubes; Stage II indicates pelvic extension; Stage III reflects peritoneal metastasis outside the pelvis and/or regional lymph node involvement; and Stage IV represents distant metastasis (e.g., pleural effusion with positive cytology or parenchymal organ involvement). Substages (e.g., IA–IC, IIIA–IIIC, IVA–IVB) denote increasing tumour spread and anatomical involvement.

**Table S5: NTA-derived extracellular vesicle characteristics across sample groups**

| **Group** | **n** | **Concentration (particles/mL)**  **mean ± SD** | **95% CI** | **Mean size (nm)**  **mean ± SD** | **95% CI** | **Mode size (nm)**  **mean ± SD** | **95% CI** |
| --- | --- | --- | --- | --- | --- | --- | --- |
| OVCA_HGSOC | 11 | 1.74 × 10¹⁰ ± 1.12 × 10¹⁰ | (1.07–2.42 × 10¹⁰) | 132.1 ± 17.7 | (121.6–142.6) | 106.0 ± 20.0 | (94.2–117.8) |
| OVCA_LGSOC | 5 | 2.82 × 10¹⁰ ± 1.78 × 10¹⁰ | (1.26–4.38 × 10¹⁰) | 141.4 ± 17.6 | (126.1–156.7) | 114.6 ± 25.3 | (92.5–136.8) |
| Mucinous | 5 | 2.82 × 10¹⁰ ± 2.16 × 10¹⁰ | (0.94–4.70 × 10¹⁰) | 136.9 ± 13.0 | (125.5–148.3) | 114.1 ± 18.0 | (98.3–129.9) |
| Endometrioid | 9 | 3.95 × 10¹⁰ ± 1.84 × 10¹⁰ | (2.72–5.18 × 10¹⁰) | 146.1 ± 13.4 | (137.5–154.7) | 115.4 ± 23.5 | (100.0–130.8) |
| Borderline | 6 | 2.07 × 10¹⁰ ± 9.30 × 10⁹ | (1.32–2.82 × 10¹⁰) | 138.4 ± 16.2 | (125.5–151.3) | 109.9 ± 16.4 | (96.8–123.0) |
| Benign | 22 | 2.58 × 10¹⁰ ± 1.55 × 10¹⁰ | (2.00–3.16 × 10¹⁰) | 133.6 ± 14.7 | (127.5–139.7) | 105.6 ± 17.8 | (98.2–113.0) |
| Healthy | 38 | 3.02 × 10¹⁰ ± 2.20 × 10¹⁰ | (2.34–3.70 × 10¹⁰) | 128.6 ± 7.8 | (126.0–131.2) | 97.6 ± 8.5 | (94.7–100.5) |

Overall statistical comparison (Kruskal–Wallis)

| **Variable** | **H statistic** | **p-value** | **Interpretation** |
| --- | --- | --- | --- |
| Concentration | ~12.8 | **0.046** | Significant differences across groups |
| Mean size | ~9.2 | 0.16 | Not significant |
| Mode size | ~10.5 | 0.10 | Not significant |

**Table S6: Summary of statistical power across biomarker panels for pairwise classification analyses**

| **Biomarker panel** | **Number of comparisons (n)** | **Mean power (%) ± SD** | **Median (IQR) (%)** | **Range (%)** | **≥80% power, n (%)** |
| --- | --- | --- | --- | --- | --- |
| EV lipids + CA-125 | 24 | 52.3 ± 36.2 | 44 (17–100) | 5–100 | 9 (37.5%) |
| EV lipids | 24 | 31.0 ± 28.6 | 26 (10–37) | 5–100 | 2 (8.3%) |
| CA-125 | 24 | 76.6 ± 30.5 | 100 (50–100) | 15–100 | 16 (66.7%) |
| Plasma lipids + CA-125 | 24 | 59.0 ± 34.7 | 53 (33–100) | 5–100 | 10 (41.7%) |
| Plasma lipids | 24 | 42.5 ± 37.4 | 25 (10–73) | 5–100 | 6 (25.0%) |

Table S6: Power (%) was calculated for each pairwise comparison across biomarker panels, including EV-derived lipids, plasma lipids, CA-125, and their combinations . Summary statistics are presented as mean ± standard deviation (SD), median with interquartile range (IQR), and range. High statistical power was defined as ≥80%.
