# Supplementary material for: Small Extracellular Vesicle and Total Lipid Profiles Provide a Diagnostic Advantage Over CA‐125 Across Ovarian Cancer Subtypes
Source: J Extracell Vesicles. 2026 Sep 1;15(9):e70348. doi: 10.1002/jev2.70348 (PMC13533800; doi:10.1002/jev2.70348)
Supplement: Supplementary file 2 [file JEV2-15-e70348-s010.docx]

**Small Extracellular Vesicle and Total Lipid Profiles Provide a Diagnostic Advantage Over CA-125 Across Ovarian Cancer Subtypes**

Shikha Rani^1,2^, Dominic Guanzon^1,2^, Andrew Lai^1,2^, Katherin Scholz-Romero^1,2^, Lewis Perrin^3^, Rohan Lourie^3^, Vaibhavi Joshi^4^, Amy E McCart Reed^4^, Kaltin Ferguson^3^, Aase Handberg^5^ Andreas Möller^6,7^, Carlos Salomon^1,2^

^1^ Translational Extracellular Vesicles in Obstetrics and Gynae-Oncology Group, Frazer Institute, Faculty of Health, Medicine and Behavioural Sciences, The University of Queensland, Herston, QLD 4006, Australia. ^2^UQ Centre for Extracellular vesicle Nanomedicine, Faculty of Health, Medicine and Behavioural Sciences, The University of Queensland, Herston, QLD 4006, Australia. ^3^Mater Research Institute, University of Queensland, Translational Research Institute, Woolloongabba, QLD, Australia. ^4^UQ Centre for Clinical Research, Faculty of Medicine, The University of Queensland, Herston, QLD 4029, Australia. ^5^Department of Clinical Biochemistry, Aalborg University Hospital, Aalborg 9220, Denmark. ^6^ Department of Otorhinolaryngology, Faculty of Medicine, The Chinese University of Hong Kong, Shatin, Hong Kong SAR. ^7^ JC STEM Lab, Li Ka Shing Institute of Health Sciences, Faculty of Medicine, The Chinese University of Hong Kong, Hong Kong SAR.

**SUPPLEMENTAL MATERIAL AND METHODS**

**Bligh-Dyer method**

Each precleared plasma and sEV sample (100 μL) was mixed directly with the 1.4 mL aliquot of ice-cold chloroform/methanol mixture (1:1, v/v) in a clear glass tube. The sample mixture was thoroughly vortexed and kept on ice for 30 min incubation. Further, 600 μL water was added to each tube and samples were vortexed and incubated on ice for 10 minutes. Further, samples were centrifugedat 2000xg for 5 minutes at 4°C. The bottom organic layer was collected into new glass tube. The upper aqueous layer was transferred into new glass tube and re-extracted with 1 ml of solvent mixture of chloroform/MeOH (1:1, v/v), following the above extraction process. The organic bottom layers containing lipids were combined and nitrogen dried at room temperature. [1].

**Folch method**

Each 100 μL precleared plasma and sEV samples were mixed with 1 mL aliquot of ice-cold chloroform/methanol mixture were added directly to the glass tubes containing plasma samples in ratio of (2:1, v/v). Samples were then vortexed and kept for 30 minutes incubation on ice. The 400 μL of MS grade water was added, vortexed and incubated on ice for another 10 minutes. The samples were centrifuged at 2000xg for 5 minutes at 4°C. The bottom organic layer was collected into new glass tube, and the aqueous layer was re-extracted with 1 mL of ice-cold chloroform/methanol mixture (2:1, v/v) using above process. Further, the organic layers were combined and dried under nitrogen at room temperature. [1].

**Matyash or MTBE method**

Each 100 μL precleared plasma and sEV samples was added to the 4.2mL aliquot of ice-cold methyl-tert-butyl ether (MTBE)/MeOH mixture was added directly to the glass tube in the ratio of 2:1, v/v. The sample tubes were then vortexed and incubated on ice for 30 minutes.A 1 mL MS-grade water was added to each tube, vortexed and incubated for additional 10 minutes. The upper organic layer was collected into new glass tube after centrigugation at 2000xg for 5 minutes at 4°C and aqueous layer was further re-extracted with 1ml mixture of MTBE/methanol/water (10:3:2.5:1 v/v) using above process. Organic layers were combined and dried under nitrogen at room temperature. [1].

**BUME method**

Each 100 μL precleared plasma and sEV samples was added to 900 μL aliquot of ice-cold mixture of Butanol/methanol in 1:1, v/v ratio was added directly to the plasma sample. Further, samples were vortexed followed by sonication on ice for 1 hour. Samples were then centrifuged at 5000x g for 1 hour and supernatant was collected into new glass tube. Supernatant was dried under nitrogen at room temperature. [1].

The dried lipids obtained from each LLE methods were reconstituted into 100ul of 100% ethanol and vortexed for 2 minutes followed by 10 minutes of sonication. To avoid the non-lipid co-extractives, samples were then centrifuged at 3000xg for 10 minutes prior transferring into MS tubes and sent for lipid detection.

**References**

1.	Apffel, A., L. Zhao, and M.J. Sartain, *A Novel Solid Phase Extraction Sample Preparation Method for Lipidomic Analysis of Human Plasma Using Liquid Chromatography/Mass Spectrometry.* Metabolites, 2021. **11**(5).
