# Supplementary material for: Small Extracellular Vesicle and Total Lipid Profiles Provide a Diagnostic Advantage Over CA‐125 Across Ovarian Cancer Subtypes
Source: J Extracell Vesicles. 2026 Sep 1;15(9):e70348. doi: 10.1002/jev2.70348 (PMC13533800; doi:10.1002/jev2.70348)
Supplement: Supplementary file 3 [file JEV2-15-e70348-s018.docx]

**Supplementary Figures legends :**

**Figure S1:**LC-MS negative and positive ion total ion chromatograms(TIC) for lipid extracts from BIOIVT gender pooled A)plasma and B)plasma derived small EVs using lipid extraction methods -BD, BUME, Folch, MTBE, SPE_cartridge and SPE_plate.

**Figure S2:Principal component Analysis(PCA) of lipid composition in (A) plasma and (B) EV samples (n=3) from LLE and SPE extraction methods.** Lipids were extracted from Plasma and EV samples using four LLE methods (BD,BUME, Folch, MTBE) and SPE methods (Cartridge and plate), further analyzed using untargeted LC-MS.

**Figure S3: Concentration and mode particle size of sEVs across patient groups.** A) Concentration of sEVs (Particle/mL) analyzed across seven patient groups: Control(n=38), Benign (n=22), Borderline (n=6), HGSOC (n=11), LGSOC (n=5), Endometrioid carcinoma (n=9) and Mucious carcinoma (n=5). No significant differences were observed in EV concentraton between groups. (B) Mode particle size(nm) of EVs measured in these patient groups. A statistically significant difference was only observed in Endometrioid carcinoma group when compared to control (p=0.0324). Data are presented as mean ± SEM, statistical analysis done using ordinary one way-ANOVA.

**Figure S4: Volcano plot analysis of differential lipid abundance in plasma and EVs across ovarian cancer groups, compared to control.** Plasma and EV lipids abundance in (A) Control vs Invasive OVCA (B) Control vs Borderline (C) Control vs Benign(D)Control vs HGSOC (E) Control vs LGSOC (F) Control vs Mucinous carcinoma and (G) Control vs Endometrioid carcinoma.

**Figure S5: Global structure and heatmap of lipid profiles**. Panel A: UMAP projection of the combined lipid dataset showing separation of samples by condition (control, invasive OVCA, benign and borderline) and sample type (EV vs plasma). Each point represents a sample and is coloured by clinical group, with different symbols indicating EV or plasma origin. Panel B: Heatmap of scaled lipid expression values across all samples, ordered by condition and sample type. Horizontal bars above the heatmap annotate clinical group and sample type, and numbered blocks highlight major clusters of co‑regulated lipids across disease states.

**Figure S6: Area under the precision-recall curve (AUPRC) and Calibration curve for ovarian cancer patients comparing with Control**. (A**) Control vs OVCA** -Control vs Invasive OVCA, Control vs Borderline, Control vs Benign, Control vs HGSOC, Control vs LGSOC, Control vs Mucinous and Control vs Endometrioid carcinoma. (B) **Benign vs OVCA** -Benign vs Invasive OVCA, Benign vs Borderline, Benign vs HGSOC, Benign vs LGSOC, Benign vs Mucinous and Benign vs Endometrioid carcinoma. (C) **Control_Benign** **vs OVCA** -Control+ Benign vs Invasive OVCA, Control+ Benign vs Borderline, Control+ Benign vs HGSOC, Control+ Benign vs LGSOC, Control +Benign vs Mucinous and Control+Benign vs Endometrioid carcinoma.

**Figure S7:** SHAP (SHapley Additive exPlanations) analysis identifying top predictive features contributing to EV lipids biomarker model performance distinguishing Control or benign vs Ovarian cancer patients.

**Figure S8:** SHAP (SHapley Additive exPlanations) analysis identifying top predictive features contributing to plasma lipids biomarker model performance distinguishing Control or benign vs Ovarian cancer patients.

**Figure S9. Confusion matrices for lipid and CA‑125 models**. Confusion matrices summarising classification performance for different biomarker combinations in distinguishing controls from invasive OVCA. Panels in the top row display results for models based on EV lipids plus CA‑125, EV lipids alone and plasma CA‑125 alone,respectively. The bottom panels show performance for plasma lipids plus CA‑125 and plasma lipids alone. Each matrix reports predicted versus reference class (control vs OVCA_all_best) at the chosen probability threshold, with cell counts corresponding to true positives, false positives, true negatives and false negatives, and accompanying specificity estimates at the indicated threshold.

**Figure S10: Volcano plot analysis of differential lipid abundance in plasma and EVs across ovarian cancer groups, compared to Benign.** Plasma and EV lipids abundance in (A) Benign vs Invasive OVCA (B) Benign vs Borderline (C) Benign vs HGSOC (D) Benign vs LGSOC (E) Benign vs Mucinous and (F) Benign vs Endometrioid carcinoma.

**Figure S11: Volcano plot analysis of differential lipid abundance in plasma and EVs across ovarian cancer groups, compared to Benign.** Plasma and EV lipids abundance in (A) Control+ Benign vs Invasive OVCA (B) Control+ Benign vs Borderline (C) Control+ Benign vs HGSOC (D) Control+ Benign vs LGSOC (E) Control +Benign vs Mucinous and (F) Control+ Benign vs Endometrioid carcinoma.

**Figure S12:** SHAP (SHapley Additive exPlanations) analysis identifying top predictive features contributing to plasma lipids and EV lipids biomarker model performance in Ovarian cancer patients with CA125 levels <35 U/mL.

**Figure S13: Area under the precision-recall curve (AUPRC) and Calibration curve for ovarian cancer patients with CA125 levels <35U/mL.** A) **Control vs OVCA** -Control vs Invasive OVCA, Control vs Borderline, Control vs Benign, Control vs Mucinous and Control vs Endometrioid carcinoma. (B) **Benign vs OVCA** -Benign vs Invasive OVCA, Benign vs Borderline, Benign vs Mucinous and Benign vs Endometrioid carcinoma. (C) Control+ Benign vs OVCA -Control+ Benign vs Invasive OVCA, Control+ Benign vs Borderline, Control+ Benign vs Mucinous and Control+ Benign vs Endometrioid carcinoma.

**Figure S14:** A) Overlaid ROC curve analysis and performance of biomarker combinations comparing Borderline patients with Ovarian cancer patients. A) Borderline vs Invasive OVCA, Borderline vs HGSOC, Borderline vs LGSOC, Borderline vs Mucinous and Borderline vs Endometrioid carcinoma. (B)Table summarizing sensitivity and specificity (95%CI), Accuracy and AUROC for each combination (C) Volcano plot analysis of differential lipid abundance in plasma and EV in respective comparisons. (D) Overlaid ROC curve analysis of biomarker performance in Ovarian cancer patients with CA125 levels <35 U/mL compared to Borderline patients (E)Table summarizing sensitivity and specificity (95%CI), Accuracy and AUROC for respective combinations.

**Figure S15.** **Quality assessment of synthetic cohorts generated for differential diagnosis and screening simulations.** (A) Density distribution of log2-transformed, scaled and centred lipid expression values in the real dataset. (B) Corresponding density distribution in the synthetic dataset, demonstrating close agreement with the real data marginal distributions. (C) UMAP projections of real (coloured points) overlaid on synthetic samples (grey points) for EV lipids and plasma lipids across differential diagnosis and screening cohorts, colour-coded by condition (benign, borderline, control, endometrioid carcinoma, LGSOC, mucinous carcinoma, HGSOC). Overlap between real and synthetic samples supports the preservation of the multivariate structure of the original data.
